# Current exercise-based rehabilitation impacts on poststroke exercise capacity, blood pressure, and lipid control: a meta-analysis

**DOI:** 10.3389/fcvm.2025.1457899

**Published:** 2025-03-24

**Authors:** Md. Moneruzzaman, Zhiqing Tang, Xiaohe Li, Weizhen Sun, Kellina Maduray, Meiling Luo, Manzur Kader, Yonghui Wang, Hao Zhang

**Affiliations:** ^1^ School of Rehabilitation, Capital Medical University, Beijing, China; ^2^ Beijing Bo’ai Hospital, China Rehabilitation Research Center, Beijing, China; ^3^ Cheeloo College of Medicine, Shandong University, Jinan, Shandong Province, China; ^4^ Department of Rehabilitation Medicine and Physical Therapy, Qilu Hospital of Shandong University, Jinan, Shandong Province, China; ^5^ Department of Cardiology, Qilu Hospital of Shandong University, Jinan, Shandong Province, China; ^6^ Department of Medicine, Solna, Clinical Epidemiology Division, Karolinska Institutet, Stockholm, Sweden

**Keywords:** stroke, exercise, blood pressure, lipid profile, neurocardiology

## Abstract

**Objectives:**

This systematic review aimed to evaluate the impact of post-stroke exercise-based rehabilitation programs on blood pressure, lipid profile, and exercise capacity.

**Methods:**

Through a systemic search of literature from inception to 2024 using five databases, we analyzed data on the mean difference (MD) using a meta-analysis method to estimate effectiveness.

**Results:**

Thirty-seven randomized control trials were included encompassing various exercises such as aerobic, resistance, stretching, exergaming, robot-assisted training, and community-based training. Significant improvement was illustrated at discharge in systolic [MD 2.76 mmHg; 95% confidence interval (CI) −1.58 to 3.92, *P* < 0.05] and diastolic (MD 1.28 mmHg; 95% CI 0.54–2.12, *P* < 0.05) blood pressure and peak oxygen volume (MD −0.29 ml/kg/min; 95% CI −0.53 to 0.05, *P* < 0.05). We also observed significant improvement at discharge in high-density lipoprotein only after resistance exercise from two articles and low-density lipoprotein only in the intervention groups compared to the control groups from ten articles.

**Conclusion:**

Overall, current exercise-based rehabilitation programs significantly improve blood pressure and exercise capacity in patients with stroke at discharge. However, lipoprotein changes remained inconclusive. Although ameliorative changes were noted in most variables, more research is needed to determine optimum exercise intensity, type combination, and health education to reduce post-stroke complications and mortality.

**Systematic Review Registration:**

https://doi.org/10.17605/OSF.IO/X89FW.

## 1 Introduction

After a stroke, 75% of patients develop cardiac diseases such as coronary artery disease, myocardial infarction (MI), atrial fibrillation (AF), heart failure (HF), and cardiac dysrhythmias (1–3). Schneck (4) stated that 19% of patients complained of heart problems just 3 months after a stroke, even though they had no history of heart disease. Several studies also illustrated that cardiovascular disease increased the risk of death after a cerebrovascular accident (4, 5). Among other cardiac cases, coronary stenosis (50%) and MI (3%) were more frequent after stroke (6). Moreover, ventricular arrhythmias, acute MI, HF, and cardiac death can be found among 4.1% of hospitalized patients with intracerebral hemorrhagic stroke, while it increases to 9% among subarachnoid hemorrhagic stroke patients (1). These post-stroke cardiac episodes are caused by stroke-induced heart damage, often known as stroke heart syndrome (7). Therefore, cardiac problems can also occur as a compensatory mechanism for stroke, known as neurogenic stress cardiomyopathy (NSC). Common manifestations of NSC are abnormal electrocardiogram (ECG) waves, ventricular wall abnormalities, and the release of troponin, a cardiac muscle regulator protein (8). Besides NSC, Takotsubo cardiomyopathy is another factor that impairs psychological stress by weakening the heart muscle after a stroke (9). However, cardiac diseases can also develop due to long-term physical inactivity and a sedentary lifestyle (5).

Consequently, 20% of ischemic strokes occur due to several cardiac complications, making cardiac diseases the most common risk factor for stroke (7, 10). When other risk factors, such as hypertension, diabetes, and smoking, are taken into account, people with AF increase their risk of stroke by approximately 5% (10). Moreover, recent studies indicate that approximately 25% of stroke patients without a prior history of AF may develop asymptomatic AF due to cardio-neurogenic mechanisms, increasing stroke recurrence risk and raising mortality by 60% (11). Fortunately, serial 12-lead ECG monitoring within the first month of post-stroke can significantly improve AF detection. However, focusing on persistent sinus rhythm and precise differentiation between AF and ventricular tachycardia are crucial to avoid further risk (12, 13). Evidence from a cohort study demonstrated that after rheumatoid heart disease, 5.2% of the patients had an incidence of stroke (14). Lackland and colleagues (15) found that cardiovascular risk factor prevention was one of the main reasons behind the decline of stroke mortality from third to fourth in the United States. Thus, cardiovascular risk factors prevention after a stroke event is inevitable.

Meanwhile, post-stroke rehabilitation comprises a variety of exercises (muscle strengthening and stretching, mobility training) and education (health education, personal grooming) to improve patients’ physical, cardiorespiratory, and cognitive performance (16, 17). Post-stroke blood pressure (BP), cardiac output (CO), heart rate (HR), and heart rate variability (HRV)—the fluctuation between two R waves—levels are essential to determine overall cardiac health and risk of stroke recurrence after rehabilitation (18, 19). Patients with depressed HVR have a lower performance rate, influencing overall recovery (20). A study on 103 subacute stroke patients found an adverse functional outcome following low HRV (18). Studies found that post-stroke cardiorespiratory fitness is not related to the factors causing stroke but to cardiovascular and pulmonary disease (21). The volume of oxygen peak (VO_2peak_), a measure of cardiorespiratory fitness, drops nearly 50% within a week of a stroke event compared to healthy individuals; although, stroke survivors’ often require a higher aerobic capacity to do routine work because of disability (22, 23). The walking ability of stroke survivors also declines due to low VO_2peak_ (22).

Previous meta-analysis studies mainly focus on the impact of aerobic exercise on post-stroke peak oxygen uptake and walking distance; evidence on the effects of post-stroke rehabilitation on cardiac variables and lipid profile was less explored (23, 24). Some meta-analyses illustrated the impact of aerobic exercise on BP and cholesterol levels, but the overall findings were inconclusive due to methodological errors among included studies and outcome measures (20, 25). Furthermore, a meta-analysis conducted by Boulouis and colleagues (26) demonstrated that lowering blood pressure after intracerebral hemorrhage was safe but unrelated to patients’ functional outcomes, which debriefed the relation between functional outcomes and cardiac variables after stroke. However, the impact of all types of rehabilitation protocols in intra- and inter-groups and comparing baseline and post-intervention changes on blood pressure, lipid profile, and functional and exercise capacity may provide insight into post-stroke rehabilitation and suggest guidelines to reduce post-stroke complications and mortality.

Therefore, our study aimed to summarize the available evidence on the effect of post-stroke rehabilitation on BP, HR, and CO, lipid profile such as HDL, and low-density lipoprotein (LDL) by comparing post-treatment changes from baseline, as well as changes between control and intervention group. The primary outcomes of our study are BP, lipid profile variables, and exercise capacity (VO_2peak_), and the secondary outcome is functional capacity (walking).

## 2 Method

This study followed the Preferred Reporting Items for Systematic Reviews and Meta-Analysis (PRISMA) guidelines (27). The protocol of this review is registered and made public in the open science forum (OSF) platform (https://doi.org/10.17605/OSF.IO/X89FW).

### 2.1 Search strategies and selection of studies

Following PICOS (28, 29) (population, intervention, comparison, outcome, and study design) methodology, a search was conducted in five online databases (Web of Science, PubMed Central, PEDro, Cochrane Library, and Scopus) for studies that reported any of our study variables such as hemodynamic changes, physical function, and cardiorespiratory properties after post-stroke rehabilitation published from inception to June 2024. For PICOS, the population consisted of all patients participating in the post-stroke rehabilitation program. Interventions included any post-stroke rehabilitation program, including exercise and health education. Studies compared the intervention effects on any variables related to cardiovascular or cardiorespiratory and functional changes after the intervention, comparing baseline and post-intervention changes. The reported study outcomes were any of our study variables such as hemodynamics (BP, HR, and CO), lipid profile variables (HDL and LDL), exercise capacity measured by VO_2peak_, and functional capacity measured by the 6 min walk test (6MWT). The study design was a randomized control trial (RCT). There was no language restriction on search engines.

The following keywords and medical subject headings (MeSH), and an asterisk (*), to identify associated keywords were utilized for a wide range of search results, such as “Cardi*,” “rehab*,” “Cerebr*,” “Heart (MeSH),” “Brain (MeSH),” “Stroke (MeSH),” “Hemorrhagic (MeSH),” “Exercise (MeSH),” “training,” “ischemic,” “embolic,” “thrombotic,” and Boolean/phrase “AND” and “OR.” In addition, all relevant article reference lists, previous systematic reviews, and guidelines were screened for selection (shown in Supplementary File 1).

One author (MM) operated the search. Three authors (MM, LX-H, M-LL) screened all articles independently, limiting studies to the following inclusion criteria: (1) study subjects are from post-stroke rehabilitation, including both genders as participants; (2) studies wherein exercise or therapy or training program was performed (such as aerobic exercise, resistance training, community-based rehabilitation program, telerehabilitation, yoga, preventive education); (3) interventional studies, which evaluated the effectiveness of an intervention, with outcomes measured at baseline and post-intervention, with or without follow-up; (4) studies wherein outcome measurement was focused on hemodynamics, lipid profile, and functional and exercise capacity as a primary or secondary outcome, respectively. The exclusion criteria of studies were as follows: (1) studies on subjects having a stroke with other neurological commodities such as Parkinson's disease or Alzheimer's disease, cardiac disease and surgeries such as bypass surgery, and musculoskeletal or traumatic brain injury; (2) studies only focused on stroke without rehabilitation; (3) observational studies (e.g., cross-sectional association or correlation study), case reports, review articles, experimental or animal studies, abstracts, editors or experts’ opinions, and letters to editors; (4) unpublished study data or studies that failed to provide outcome data after contact with the author(s). Discussions with the supervising author (HZ) resolved any disputes regarding study selection.

### 2.2 Screening of article

We utilized reference manager software “Zotero” (30) and “Rayyan” (31) for study screening and finding duplicates. Titles and abstracts were screened for primary selection, and full text and data availability were assessed for final study selection. The author (MM) contacted the respective authors for data availability. Any disagreement was solved through discussion.

### 2.3 Quality assessment

For the quality of the study and the risk of bias assessment, two authors (MM and W-ZS) utilized “PEDro” (32) and the Cochrane Handbook for risk of bias assessment tool “ROB 2.0” (33). Regarding PEDro scores, studies were categorized as fair (>4), good (6–8), and excellent (9–10). The ROB 2.0 was assessed for the randomization process, deviation from the intended interventions, missing outcome data, measurement of the outcome, and selection of the reported result and categorized as low risk, some concern, and high risk. The leave-one-out forest plot checked for any ambiguity in the study data.

### 2.4 Data extraction

Two authors (MM and KM) extracted all available data independently from included studies, including first author, year, country, sample size, age, gender, inclusion criteria of participants, type of stroke and disability, rehabilitation programs (such as aerobic exercise, balance training, upper and lower limb exercise, resistance training, health education), standard rehabilitation protocol, treatment duration and intensity, treatment outcomes [BP, CO, HR, HDL, LDL, total cholesterol (TC), triglycerides (TG), FBG, VO_2peak_, time up and go (TUG), Berg balance score (BBS), 6MWT], follow-up, and summary of all findings (shown in Tables 1, 2).

**Table 1:** Summary of findings of all included articles.

Author, year, country	Total sample size, *N*	Gender, *n* (M/F)	Age (mean, SD)	Inclusion criteria on stroke incidence	Type of disability or stroke and severity among included participants, (*n*)	Treatment duration	Measure(s) and outcome(s)	Follow-up duration	PEDro score
Tang et al., 2013, Canada (49)	50	29/21	59.71, 12.35	• >1 year post-stroke	• Lacunar, 7 • Ischemic, 19 • Hemorrhagic, 16 • Unknown, 8	6 months	Aerobic capacity (VO_2peak_), arterial stiffness, functional capacity (6MWT), hemodynamic and cardiac function (left ventricular ejection fraction, trans-mitral inflow, lateral mitral annulus, right atrial emptying fraction, lateral tricuspid annulus), blood profile (TC, LDL, HDL, triglycerides, fasting blood glucose, homocysteine)	Not estimated	8/10
Kim et al., 2014, South Korea (43)	20	N/A	54, 8.98	• Stroke incidence within the last 6 months	• Unilateral stroke with hemiparesis • Oriented and able to walk 6 min with or without assistance	4 weeks	Lung capacity (FVC, FEV, PEF), functional capacity (6MWT), shortness of breath modified Borg dyspnea scale (SBMBDS)	Not estimated	4/10
Moore et al., 2014, United Kingdom (45)	40	34/6	69, 9.5	• >6 months post-stroke	• Ischemic stroke, 37 • Mild to moderate impairment (NIHSS 0–8) • Able to complete 6MWT (with/without a stick)	19 weeks	Glucose control, insulin sensitivity test, cerebral blood flow, cardiorespiratory fitness, resting blood pressure, lipid profile, body composition, physical performance on 6MWT,10MWT, Berg balance scale (BBS) test, cognitive function	Not estimated	7/10
Moore et al., 2016, United Kingdom (46)							Cardiorespiratory fitness (VO_2peak_, work rate), cardiac hemodynamics (CO, BP, cardiac power output), peripheral muscle oxygen extraction, functional capacity (6MWT, 10MWT, TUG, BBS)	Not estimated	7/10
Faulkner et al., 2016, United Kingdom (38)	47	35/12	55.94, 11.04	• Within 7 days of symptom onset	• Transient ischemic stroke or minor stroke	12 weeks	Central and peripheral blood pressures on an oscillometric device, HR, RPE	Not estimated	7/10
Gambassi et al., 2019, Brazil (39)	22	9/13	63.45, 11.86	• Stroke incidence within the last 6 months	• Able to walk with or without a walking aid	Over 8 weeks	Functional parameters (isometric handgrip of paretic and non-paretic limbs, 10MWT, 5-repetition sit-to-stand, TUG), hemodynamic parameters (BP, heart rate variability), oxidative stress markers (thiobarbituric acid reactive substances, carbonyls, NADPH oxidase, hydrogen peroxide, superoxide dismutase, and plasma nitrite analyses)	Not estimated	6/10
Hus et al., 2020, China (Taiwan) (41)	23	20/3	55.45, 4.66	• Stroke duration >24 months • Stroke events with stable clinical status >3 months	• Thrombosis, 15 • Hemorrhage, 8	3–4 months	Aerobic capacity (bicycle ergometer), cerebral oxygenation [non-invasive continuous CO monitoring, near-infrared (NIR) system], peak CO, serum brain-derived neurotrophic factor (BDNF)	Not estimated	6/10
Gjellesvik et al., 2020, Norway (40)	70	41/29	58.13, 9.15	• Three months to 5 years after the first-ever stroke	• Transient ischemic stroke or minor stroke	8 weeks	Graded exercise treadmill test (VO_2peak_), systolic and diastolic BP, blood profiles (lipid levels, insulin resistance, hemoglobin, HDL, LDL, TC, triglycerides, glycosylated hemoglobin, and C peptide), HR, lactate, minute ventilation, respiratory exchange ratio, carbon dioxide output, Borg balance scale	12 months	7/10
Tollar et al., 2020, Hungary (50)	641	349/292	66.5, 5.87	• 2–4 weeks after stroke	• Subacute ischemic stroke (326) • Walking disability was severe • Moderately severe ADL-specific disability	5 weeks	Functional capacity (6MWT), health-related QoL, HR, BP, and RPE, static balance, Berg balance scale, Beck depression scale, modified Rankin scale	Not estimated	8/10
Deijle et al., 2022, Netherlands (37)	119	70/49	64.70, 9.7	• Adult with TIA or minor ischemic stroke 1 month ago, able to walk, and no cardiopulmonary complication and chronic diseases in <2 years	• TIA (34) • Ischemic stroke (26)	12 months	Montreal Cognitive Assessment (MoCA), maximal oxygen consumption (V˙O_2max_) by cycle ergometer, ECG, hospital anxiety and depression scale (HADS), fatigue severity scale (FSS), BP, TC, LDL	24 months	8/10
Lapointe et al., 2023, Canada (44)	52	33/19	69.2, 10.7	• Ischemic stroke or TIA with a minimum of 3 months post-event. • Ambulatory capacity over 10 min without or with assistive devices	• Able to walk independently or with an assistive device	6 months	peak oxygen uptake (VO_2peak_), systolic and diastolic blood pressures, lipid profile, HbA1c, waist circumference, body composition, self-reported physical activity, functional level, anxiety and depression, and cognitive functions	12 months	4/10
Kang et al., 2023, Korea (42)	16	10/6	55.26,14.22	• Mini Mental State Examination score >22 • Ability to follow verbal instructions and communicate	• Patient with ischemic stroke and without any severe disability	8 weeks	Irisin, muscle strength, cardiorespiratory endurance, and body composition before and after the program	Not estimated	5/10
Sakakibara et al., 2022, Canada (48)	126	87/39	68.1, 9.7	• Stroke in the last 12 months • Mild to moderate stroke severity on modified Rankin scale score ranging from 1 to 4	• Ischemic (89) • Hemorrhagic (24) • Lacunar (8)	6 months	Lifestyle behavior, Health-related quality of life, depressive symptoms and cognitive function, walking physical activity, blood pressure, glycated hemoglobin, fasting glucose, high- and low-density lipoprotein, C-reactive protein, and homocysteine	12 months	8/10
Krawcyk et al., 2019, Denmark (69)	71	49/22	63.7, 9.2	• First-time lacunar stroke or a recurrent event of lacunar stroke with mild neurological symptoms on the Scandinavian stroke scale (43–58 points) • Able to speak and read	• Lacunar	3 months	Cardiorespiratory fitness, post-stroke fatigue, cognitive behavior, BP, BMI, PAS2 (questionnaire returned on assessment visit, reporting average physical activity for the past two weeks)	Not estimated	7/10
Aguiar et al., 2020, Brazil (53)	22	16/6	50, 10.46	• ≥20 years of age • Diagnosis of stroke >6 months.	• Ischemic (19) • Hemorrhagic (2) • Ischemic and hemorrhagic (1)	12 weeks	Cardiorespiratory fitness, walking distance, QoL	16 weeks	7/10
Macko et al., 2005, United States (63)	61	43/18	63.4, 9.04	• >45 years of age • Chronic stroke (>6 months) • Hemiparetic gait	• Not specified	6 months	Functional mobility, cardiovascular fitness	Not estimated	5/10
Reynolds et al., 2021, Australia (66)	20	18/2	57.5, 11.2	• Aged ≥18 years • stroke (ischemic or hemorrhagic) within the past 6 weeks (minimum) to 12 months • Able to walk at least 100 m (with or without aids or standby supervision)	• Ischemic (16) • Hemorrhagic (4)	12 weeks	Functional mobility, cardiovascular fitness, QoL		7/10
Ribeiro et al., 2017, Brazil (67)	38	23/15	58.5	• Aged between 21 and 70 years • Subacute stage (until 1 year from the onset of stroke) • Able to walk independently for 10 m • Able to understand simple motor commands	• Ischemic (32) • Hemorrhagic (6)	9 days	Blood pressure, heart rate, gait speed on treadmill	Not estimated	5/10
Sandberg et al., 2020, Sweden (68)	52	20/32	74.7,9.3	• At least 18 years • Had a first stroke • Able to perform aerobic exercise • Understand spoken and written instructions • Impairments on National Institutes of Health stroke scale (NIHSS) score of 7–42	• Ischemic (46) • Hemorrhagic (6)	3 weeks	Blood pressure, heart rate	Not estimated	6/10
Wijkman et al., 2018, Sweden (72)	53	26/27	70.9, 7.6	• 50 years and older • Ability to understand the Swedish language • Ability to walk 5 m with or without the support of any means or person • Be able to get up on a cycle ergometer and cycle at their own chosen pace • Approval of medically responsible physician to conduct physical/training in groups	• Ischemic (52) • Hemorrhagic (1)	12 weeks	Cardiorespiratory function, balance, walking capacity, QoL	6 months	6/10
Acheampong et al., 2018, Ghana (52)	13	5/8	59.88, 10.88	• Age group of 35–68 years • Diagnosed with stroke for <2 years • Cleared of severe complications (e.g., blindness, kidney, and nerve damage)	• Ischemic	10 weeks	Biochemical variables, physiological variables, and cardiovascular variables	Not estimated	4/10
Globas et al., 2012, Switzerland (55)	36	29/7	68.7, 6.3	• >60 years, • Residual hemiparetic gait was enrolled >6 months after stroke • The ability to walk on the treadmill at ≥0.3 km/h for 3 min with handrail support	• Ischemic (36)	13 weeks	Cardiorespiratory fitness, gait velocity	One year	7/10
Han et al., 2017, South Korea (56)	20	12/8	60.9, 13.2	• First-ever primary ischemic or hemorrhagic stroke • Interval between stroke onset and recruitment was ≤3 months • Presence of hemiparesis • Mild to moderate walking deficit • Ability to follow verbal instructions and communicate with investigators	• Ischemic (14) • Hemorrhagic (4)	6 weeks	Cardiorespiratory fitness, walking endurance, ADL	Not estimated	6/10
Jin et al., 2013, China (57)	128	91/37	56.96, 6.5	• Ischemic stroke (>6 months) • Independence in ambulation with or without a walking aid	• Ischemic (128)	12 weeks	Cardiorespiratory fitness, walking	Not estimated	4/10
Lee et al., 2013, South Korea (60)	16	8/8	63.25, 14.1	• An onset of stroke within 6 months • Presented with mild to moderate paresis of the lower extremities	• Ischemic (10) • Hemorrhagic (6)	4 weeks	Cardiorespiratory fitness, BP, walking ability	Not estimated	4/10
Quaney et al., 2009, United Kingdom (65)	38	17/21	61.53, 13.6	• Adult • 6 months prior • Residual hemiparetic deficits in either the upper or lower extremity • Adequate cardiac function	• Not specified	8 weeks	Exercise capacity, cognitive function, mobility	Not estimated	6/10
Sutbeyaze et al., 2008, Turkey (71)	45	24/21	61.8, 11.6	• First episode of unilateral stroke with hemiparesis during the previous 12 months • Sufficient unilateral upper torso and extremity nerve function and strength to accomplish arm crank ergometry • Ability to understand and follow simple verbal instructions	• Ischemic (33) • Hemorrhagic (12)	6 weeks	Cardiorespiratory fitness	Not estimated	7/10
Potempa et al., 1995, United States (64)	42	23/19	Not specified	• Age 21–77 years • <6 months of stroke onset	• Mild to moderate hemiparesis	10 weeks	Cardiovascular variables and fitness	Not estimated	5/10
Zou et al., 2015, China (73)	56	34/22	51.85, 7	• Aged <60 years • >6 months after stroke onset • Achieved basic functional independence • Walk independently with or without walking aids	• Ischemic (37) • Hemorrhagic (19)	8 weeks	Cardiovascular variables and blood profile	Not estimated	7/10
Stoller et al., 2015, Switzerland (70)	14	9/5	61, 11	• Older than 18 years • Less than 20 weeks of post-stroke • Ability to understand the procedures	• Ischemic (12) • Hemorrhagic (2)	4 weeks	Cardiorespiratory fitness	Not estimated	8/10
Faulkner et al., 2013, New Zealand (54)	60	31/29	68.5, 10.4	• All patients with new TIA without any other major complications such as dementia or unstable cardiac condition	• TIA (60)	8 weeks	Cardiorespiratory fitness and cardiovascular variables	12 months	8/10
Kirk et al., 2013, United Kingdom (58)	24	19/5	67.5, 9.3	• One month of stroke incidence • Able to walk with or without a stick, no history of falls within the past 2 months • Cognitive capacity sufficient to undertake group exercises	• TIA (18)	6 weeks	Cardiorespiratory fitness and cardiovascular variables	6 months	8/10
Kono et al., 2013, Japan (59)	70	48/22	63.95,9.4	• Recent onset of stroke • Modified Rankin scale score of 0–1 • Returned home directly after discharge • Without any communication disability	• Not specified	24 weeks	Cardiovascular and orthopedic risk factor	2.9 years (median)	8/10
Lennon et al., 2008, Ireland (61)	48	28/20	59.75, 10.07	• Over 18 years • 1-year post-ischemic stroke • Any level of independence	• Infract (42) • Unknown (6)	10 weeks	Cardiopulmonary fitness and blood profile	Not estimated	7/10
MacKay-Lyons et al., 2022, Canada (62)	184	121/63	65.08,10.05	• Over 17 years old. • Within 3 months of stroke onset	• Not specified	12 weeks	Cardiopulmonary fitness and QoL	6 months and 12 months	8/10

TC, total cholesterol; LDL, low-density lipoprotein; HDL, high-density lipoprotein; FVC, forced vital capacity; FEV, forced expiratory volume; PEF, peak expiratory flow; 6MWT, 6 min walking test; 10MWT, 10 min walking test; CO, cardiac output; BP, blood pressure; TUG, timed up and go; HR, heart rate; RPE, ratings of perceived exertion; NHIS, National Health Interview Survey.

**Table 2:** Characteristics of included studies rehabilitation program and summary of findings.

Study	Rehabilitation program(s)	Sample size	Age (mean ± SD)	Data analyzed	Rehabilitation protocol(s)	Treatment intensity	Treatment duration	Summary of result(s)
Tang et al., 2013_A	Aerobic exercise (AE)	25	65.9 ± 6.4	HDL, LDL, TG, TC, 6MWT, VO_2peak_, FBG	Walking (overground brisk and inclined), cycle ergometry (upright and recumbent), marching on the spot, repeated sit-to-stand, and step-ups onto platform steppers	High-intensity (40%–70%–80% of HRR)	Total 60 min, 3 times/week	• There were no significant changes in VO_2peak_ in either group (*P* = 0.45). • Improved 6MWT distance (*P* = 0.02) and reduced total LDL cholesterol and triglyceride levels. • No improvement in lipids, glucose, and homocysteine levels and ambulatory capacity
Tang et al., 2013_B	Balance and flexibility (BF)	25	66.9 ± 7.8		Stretching, weight-bearing, postural re-education, and balance exercises	Low-intensity (<40% of HRR)		
Yim et al., 2014_A	Upper and lower-body exercise with respiratory training	10	54.10 ± 11.69	6MWT	Joint mobility, eccentric contraction, muscle, strengthening, walking exercise, automated full-body workout, and respiratory training	Low-intensity	Total 60 min, 3 times/week	• Increase the mean difference in 6MWT; the walking distance before and after exercise significantly differed between groups. • Increase shortness of breath modified Borg dyspnea scale before and after exercise in both groups (*P*<0.05)
Yim et al., 2014_B	Upper and lower-body exercise training	10	53.90 ± 5.82		Walking exercise, joint mobility, eccentric contraction, muscle strengthening, automated full-body workout			
Moore et al., 2014_A	Fitness and mobility exercise	20	68 ± 8	Blood pressure, LDL, HDL, total cholesterol, 6MWT, VO_2peak_	Upper and lower limb stretching, functional strengthening, balance, agility, and fitness training	Moderate intensity, initially 40%–50% of their maximum HR, increasing increments of 10% every 4 weeks, up to 70%–80%, Strength/balance exercises were progressed by increasing repetitions and loading	45–60 min, 3 times/week	• Exercise increases work rate and peak oxygen consumption (increased by 17%). • A significant within-group increase was demonstrated in diastolic BP in the control group, but between-group differences in resting diastolic but not systolic BP in favor of exercise. • The exercise group found significant improvement in walking ability, balance, cognition, mood, strength, physical activity, and overall stroke recovery. • HDL-C levels increased significantly in the exercise group compared with controls. • Total cholesterol, LDL-C levels, body mass index, and composition were unchanged in both groups following the intervention
Moore et al., 2014_B	Education about health care and stretching	20	70 ± 11		Home-based stretching exercise and health education on medication, diet, and physical activity	Not estimated		
Moore et al., 2016_A	Fitness and mobility exercise	20	68 ± 8	TUG, CO	Upper and lower limb stretching, functional strengthening, balance, agility, and fitness training	Moderate intensity, initially 40%–50% of their maximum heart rate, increasing increments of 10% every 4 weeks up to 70%–80%, strength/balance exercises were progressed by increasing repetitions and loading	45–60 min, 3 times/week	• The exercise group significantly improved peripheral oxygen utilization but did not alter the central oxygen supply. • Exercise-induced changes in peak oxygen consumption and peripheral muscle oxygen utilization were not strongly associated with improved function. • A moderately significant correlation was observed between exercise-induced peak oxygen consumption change and 10MWT and TUG test scores
Moore et al., 2016_B	Education about health care and stretching	20	70 ± 11		Home-based stretching exercise and health education on medication, diet, and physical activity	Not estimated		
Faulkner et al., 2016_A	Aerobic exercise	25	66 ± 12	HR, BP, HDL, LDL, TG, TC, 6MWT, VO_2peak_, FBG	Continuous walking and cycling	Age-predicted 90% of maximal HR, BP, RPE	30–60 min, 2 times/week	• Exercise program soon after stroke/TIA diagnosis significantly improved cSBP (7%) and reduced AIx (15%). • A significant interaction for peripheral and central pulse pressure was also observed for cSBP, pSBP, and the exercise group presenting lower values than the control group post-intervention. • Pulse pressure was increased in the control group but decreased in the exercise group at baseline and post-intervention
Faulkner et al., 2016_B	Health education	22	68 ± 10		Education on secondary prevention and healthcare	Not estimated	Not estimated	
Gambassi et al., 2019_A	Resistance training (RT) with neurological rehabilitation	11	66.4 ± 10.1	BP, HR, TUG	Dynamic RT (seated row and squat on the chair, vertical chest presses and squat on the chair, knee extension and squat on the chair), physical movements that mimic basic and instrumental ADL, postural changes, and gait exercises on parallel bars. With an elastic belt with standard rehabilitation	Seated row and squat on the chair, vertical chest press and squat on the chair, knee extension, and squat on the chair, physical movements that mimic basic and instrumental ADL, postural changes, and gait exercises on parallel bars	40–50 min, 2 times/week	• 8-week dynamic resistance training protocol with elastic bands improved physical function, hemodynamic parameters, autonomic modulation, and oxidative stress markers in chronic ischemic stroke survivors. • A significant reduction in upper-limb muscle strength (i.e., IHGPL and IHGNPL) was observed in the control group. • Improved 10MWT, sit-to-stand, TUG value. • SBP and DBP remained unchanged in both groups over the experimental period. • Heart rate and DP were significantly reduced in the training group after 8 weeks
Gambassi et al.2019_B	Neurological rehabilitation	11	60.5 ± 13.2		Standard rehabilitation	Physical movements that mimic basic and instrumental ADL, postural changes, and gait exercises on parallel bars		
Hus et Aa., 2020_A	Aerobic exercise	10	58.5 ± 4.35	CO, VO_2peak_	Cycling, balance, range of motion, or therapeutic exercise with high-intensity interval training (HIIT)	3 min intervals at 80% VO_2peak_,3 min of exercise at 40% of VO_2peak_, cooldown at 30% VO_2peak_	45–60 min, 2–3 times/week	• VO_2peak_, CO, dendritic growth (*P* = 0.017), and serum BDNF levels increase significantly after HIIT than MICT. • VO_2peak,_ was positively correlated with deoxyhemoglobin (*r* = 0.634, *P* = 0.006) and %neurites (*r* = 0.551, *P* = 0.041)
Hus et al., 2020_A	Aerobic exercise	13	53.1 ± 3.45		Cycling, balance, range of motion, or therapeutic exercise with moderate-intensity continuous training (MICT)	60% of peak oxygen consumption (VO_2peak_)		
Gjellesvik et al., 2020_A	Walking, home advice about physical activity, health education	36	57.6 ± 9.2	BP, HDL, LDL, TG, VO_2peak_	Treadmill exercise 4 × 4 HIITT (speed increased by 0.5–1.0 km/h^−1^, inclination increased by 1%–2%)	85%–95% of peak HR, 3 min of active recovery at 50%–70% of peak HR	38 min, 3 times/week, 8 consecutive weeks	• The HIIT group's improvement in functional capacity and VO_2peak_ after 8 weeks was higher than the standard care group post-intervention but did not maintain during the follow-up period assessment
Gjellesvik et al., 2020_B	Home advice about physical activity and health education	34	58.7 ± 9.2		Physically active and engaging in activities with moderate to high intensity	Not estimated	3–5 days per week	
Tollar et al., 2020_A	Twice/day high-frequency and high-intensity exercise	286	67.6 ± 5.49	BBS	EX1: double exergaming exercise (EX), exergaming used three modules of the Xbox 360 core: system reflex ridge, space pop, just dance, agility training, and medical massage of the lower limb	RPE of 14–16 of 20	60 min, 2 times/day (5 consecutive sessions, 5 weeks, 50 sessions)	• HIIT exergaming twice or once daily significantly improves over low-intensity standard care on clinical and motor symptoms, BP, and QoL. • QoL, Barthel index, BBS, 6MWT, and standing posture improved significantly in the EX2 group and the same in the EX1 and control groups. • Systolic and diastolic resting BP decreased more in the EX2 and EX1 groups than in the control group
Tollar et al., 2020_B	Once a day high-frequency and high-intensity exercise	272	65.9 ± 6.10		EX2: single exergaming exercise, exergaming used three modules of the Xbox 360 core: system reflex ridge, space pop, just dance, agility training, and medical massage of the lower limb			
Tollar et al., 2020_C	Standard care	83	177.9 ± 4.23		Group exercises include sitting, walking, and balance exercises, as well as upper extremity and trunk muscle strength exercises by lifting, lowering, and rotating medicine balls and end-weighted sticks. Exercises standing target lower extremity function (stepping variations such as forward, backward, diagonally while standing on 1 leg), weight shifting, coordinative movements with arms while walking with and without various sensory implements, and squatting movements with arm support on a chairback to strengthen the lower extremity extensor mechanism, medical massage of the lower limb		60 min, once a day	
Lapointe et al., 2023_A	MICT	16	65.6 ± 11.3	VO_2peak_, SBP, DBP, HR, HDL, LDL	Supervised aerobic exercise (walking, swimming, dancing, or cycling)	RPE at 4–6/10; 50% peak power output (PPO)	20–40 min with 5 min warmup and 5 min cooldown	• A 6-month HIIT + MICT combined program and a standard MICT program induced similar improvements in CRF, and self-reported physical activity compared with a control group
Lapointe et al., 2023_B	HIIT + MICT	19	71.8 ± 9.9		MICT exercises with increased bouts 10 min	RPE at 4–6/10; 95% of PPO		
Lapointe et al., 2023_C	Usual care	17	69.6 ± 10.7		Standard exercise program	Not estimated		
Deijle et al., 2022_A	MoveIT training	60	64.7 ± 8.9	VO_2peak,_ SBP, DBP, TC, LDL	10 participants group-wise supervised aerobic exercise (cycle ergometer, treadmill, rowing)	40%–80% of targeted heart rate, RPE 11–16, and Borg scale 6–20	30 min, 2–3 times/week	• No benefit was found among participants in terms of cardiorespiratory function, but the experimental group was found to be more fatigued than the usual care group
Deijle et al., 2022_B	Standard care	59	63.9 ± 10.6		Usual care according to institutional guideline	Not specified	Not specified	
Kang et al., 2023_A	Community-based rehabilitation	6	54.33 ± 18.22	6MWT, VO_2peak_	Strength training (both limbs), cardiovascular exercise (high knee, sidestep, pogo jump, jumping jack, front step, back step, and knee up), and game-based leisure-time physical activities	65%–80% of the individual's maximum heart rate	60 min, 3 times/week	• Community-based exercise improved leg and trunk strength, peak oxygen consumption values, and body composition, which suggested that it might be an effective intervention to increase irisin levels and prevent a stroke-related decline in muscle function
Kang et al., 2023_B	Standard care	10	56.20 ± 9.64		Usual care according to institutional guideline	Not specified	Not specified	
Sakakibara et al., 2022_A	Stroke coach	64	67.2 ± 9.2	SBD, DBP, HDL, LDL, FBG	Monitored lifestyle through telephone calls; participants used self-management health monitoring cards and a self-monitoring kit	Not estimated	30–60 min telephone call with 5–10 min check-in calls	• Stroke coach did not improve lifestyle behavior, but memory training improved HRQoL and glucose control among community-living stroke survivors with mild stroke-related disability
Sakakibara et al., 2022_B	Memory training	62	69.1 ± 10.2		Monitored patients’ daily activity and trained them on cognitive health management			
Krawcyk et al., 2019_A	Aerobic exercise and home-based HIIT	31	63.7 ± 8.9	BP, LDL, HDL, TC, TG	Aerobic exercise with HIIT and medical education	77%–93% of the maximum HR, 14–16 on the Borg-rated PER	15 min of home-based HIIT, 3X3 continuous bouts, and 2 min active recovery	• Within 3 months, HIIT did not have a superior effect on cardiorespiratory fitness
Krawcyk et al., 2019_B	Aerobic exercise	32	63.7 ± 9.2		Aerobic exercise and medical education	Self-regulated	Self-regulated	
Aguiar et al., 2020_A	Treadmill walking	10	52, 11	VO_2peak_, 6MWT	Aerobic training with a progressive increase in speed	60%–80% HRR	40 min	• Treadmill or outdoor-overground walking does not have any significant impact on physical activity levels but improves depression, endurance and mobility. • Treadmill walking improves QoL
Aguiar et al., 2020_B	Over the ground walking	8			Over the ground walking	≤40% HRR	40 min	
Macko et al., 2005_A	Walking on treadmill	26	63,10	VO_2peak_, 6MWT	Treadmill aerobic exercise at minimum 40%–50% of HRR increased progressively 5% HRR every 2 weeks	60%–70% HRR	40 min	• Treadmill aerobic exercise improves functional mobility and cardiovascular fitness
Macko et al., 2005_B	Usual care and stretching	20	64,8		Usual care, stretching, and low-intensity treadmill walking are at 30%–40% HRR	30–40% HRR	35 min	
Reynolds et al., 2021_A	Progressive moderate intensity	10	54.6, 8.9	VO_2peak_, 6MWT	Progressive, moderate-intensity training with upright stationary cycle ergometer, recumbent bike, treadmill, upper-limb ergometer, stepper, cross-trainer, and stairs	Minimum at 40% of HRR increased by 5% HRR as tolerated on RPE 11–13	30 min	• Moderate-intensity cardiovascular fitness training is safer for stroke survivors than usual care. But does not have any superior effect than the standard care
Reynolds et al., 2021_B	Standard care	10	60.3, 12.9		Upright stationary cycle ergometer (maximum 5 min per session), walking in rails or gym, standing balance, basic strengthening (slow squats, seated quadriceps extension), and bed-based exercises	<40% HRR; RPE <11		
Ribeiro et al., 2017_A	Treadmill walking with ankle load	19	57	SBP, DBP, HR	Gait training on a treadmill with added load to the non-paretic lower limb	50% of HR maximal, ankle load equivalent to 5% of the 234 body weight	30 min	• An additional load on non-paretic lower limbs with gait training does not alter cardiovascular parameters and can be considered useful and safe for stroke patients
Ribeiro et al., 2017_B	Treadmill walking	19	60		Gait training on a treadmill	Unweighted and at a tolerated range		
Sandberg et al., 2020_A	In-bed cycling	23	72.7,12	SBP, DBP, HR	An electrical in-bed cycling with a maximum of 15 sessions, each participant was encouraged to cycle by himself/herself, but otherwise, the cycle was able to run passively at 20 revolutions per minute	RPE (11–13) ≥50% of maximum oxygen uptake and HRR maximum of 60%	20 min	• Aerobic in-bed cycle exercise has a significant impact on normalizing blood pressure response to exercise
Sandberg et al., 2020_B	Usual care	29	76.3,6.4		Standard care	Not specified	Not specified	
Wijkman et al., 2018_A	Aerobic exercise	29		SBP, DBP, HR	15 min warmup (phase one), an 8 min aerobic part on an ergometer cycle (phase two), a 10 min part with low-intensity mixed exercises (phase 3), another 8 min aerobic part on an ergometer cycle (phase 4), and a final 15 min cooldown (phase 5)	Not specified	60 min	• Stroke patient usually has an exaggerated SBP response. Aerobic exercise improves aerobic capacity, walking ability, balance, and self-reported quality of life
Wijkman et al., 2018_B	Usual care	24			Only general advice about physical exercise and activity, no specific exercise program	Not specified	Not specified	
Acheampong et al., 2018_A	Combined exercise	5	Not estimated	SBP, DBP, LDP, HDL, TG, TC, HR	Aerobic exercise, flexibility exercise, resistance training	40%–70% of HRR	60 min	• The combined exercise group showed significantly improved BP and lipids in post-intervention results compared to the pre-treatment
Acheampong et al., 2018_B	Conventional exercise	8	Not estimated		Activities in daily living, not any specific exercise	Not specified	Not specified	
Globas et al., 2012_A	Aerobic treadmill exercise	18	68.6 ± 6.7	VO_2peak_, 6MWT, BBS	Treadmill exercise	60%–80%	10–45 min	• Aerobic treadmill exercise with a training intensity just below the anaerobic threshold improves chronic stroke survivors’ cardiovascular fitness, gait, balance, mobility, and quality of life in older persons
Globas et al., 2012_B	Usual care	18	68.7 ± 6.1		Passive, muscle tone–regulating exercises, balance training	Not specified	60 min	
Han et al., 2017_A	Land-based exercise	10	62.40 ± 12.72	6MWT, VO_2peak_, HR	Land-based aerobic exercise program using upper and lower-body ergometers with standard care includes stretching and strengthening exercises of the upper extremities and task-oriented therapy	Not specified	50 min, 5 times/week	• Patients with subacute stroke who are able to complete 6 weeks of aquatic treadmill significantly improve walking endurance and cardiorespiratory fitness than land-based exercise
Han et al., 2017_B	Aquatic treadmill exercise	10	59.40 ± 14.25		Water-based aerobic exercise on a motorized aquatic treadmill with standard care includes stretching and strengthening exercises of the upper extremities and task-oriented therapies	50%–85% HRR	50 min, 5 times/week	
Jin et al., 2013_A	Aerobic cycling training	65	57.6 ± 6.6	SBP, DBP, 6MWT, VO_2peak_, HR	Patients pedaled for 6–10 min in each task condition, and 2–3 min of rest were provided between each task with control group training	50%–70% HRR	40 min/day; 5 times/week	• Aerobic cycling training improves cardiovascular fitness in patients with chronic stroke
Jin et al., 2013_B	Usual care	63	56.3 ± 6.5		Supervised stretching movements lasting 35 min and 5 min low-intensity overground walking training	20%–30% HRR	35 min/day; 5 times/week	
Lee et al., 2013_A	Functional electrical stimulation	8	63.25 ± 15.00	SBP, DBP, 6MWT, BBS, VO_2peak_, HR	Electrical stimulation on the paretic quadriceps, hamstring, gluteus maximus, and tibialis anterior muscles using two EMG during assistive ergometer training	A pedaling cadence of 30 rpm for 30 min	5 times/week	• Assisted ergometer training with an FES increases subacute stroke patients’ aerobic capacity
Lee et al., 2013_B	Assistive ergometer training	8	63.25 ± 14.12		assistive ergometer training			
Quaney et al., 2009_A	Aerobic exercise	19	64.10 ± 12.30	VO_2peak_, BBS	Stationary bike	70% HRR	45 min/day; 3 times/week	• Aerobic exercise improves mobility and some cognitive functions related to motor learning
Quaney et al., 2009_B	Streaming exercise	19	58.96 ± 14.68		Upper and lower limb stretching exercise	Not specified	45 min/day; 3 times/week	
Sutbeyaze et al., 2008_A	Breathing retraining	15	60.8 ± 6.8	VO_2peak_	15 min of diaphragmatic breathing combined with pursed-lips breathing, followed by 5 min of air-shifting techniques and 10 min of voluntary isocapnic hyperpnea. The patients had a 5 min interval before each type of exercise with conventional rehabilitation	Not specified	15 min/day; 6 times/week	• Respiratory muscle training program has a significant short-term impact on respiratory muscle function
Sutbeyaze et al., 2008_B	Inspiratory muscle training	15	62.8 ± 7.2		Inspiratory muscle training with conventional rehabilitation	40%–60% of maximum inspiratory pressure	15 min/day; 6 times/week	
Sutbeyaze et al., 2008_C	Usual care	15	61.9 ± 6.15	BP, HR, VO_2peak_	Conventional rehabilitation	Not specified	5 times/week	
Potempa et al., 1995_A	Aerobic exercise	19	Not specified		Aerobic exercise training	Not specified	30 min/day; 3 times/week	• Aerobic exercise improves submaximal SBP and aerobic capacity
Potempa et al., 1995_B	Passive ROM exercise	23	Not specified		Passive ROM exercise of upper and lower limb	Not specified	30 min/day; 3 times/week	
Zou et al., 2015_A	Resistance training	28	52.3 ± 6.9	BP, HR, LDL, HDL, FBG	Three sets of 15 unilateral repetitions of the leg press, leg extension, and leg curl movements on the training machines	Not specified	40 min/day; 3 times/week	• Resistance training has a significant role in improving hyperglycemia and dyslipidemia
Zou et al., 2015_B	Usual care	28	51.4 ± 7.2		Three sets of 15 unilateral repetitions of the leg press, leg extension, and leg curl movements on the training machines	Not specified	40 min/day; 3 times/week	
Stoller et al., 2015_A	Feedback-controlled robotics-assisted treadmill exercise (FC-RATE)	7	57 ± 12	VO_2peak_	Progressive cardiovascular exercise using FC-RATE with usual care, including physical, occupational, and speech and language therapy	40%–70% HRR	30–60 min/day; 4–5 times/week	• FC-RATE and conventional RATE significantly increase cardiopulmonary performance and exercise intensity, but recommended intensity levels for cardiovascular training are not consistently achievable
Stoller et al., 2015_B	Robotics-assisted treadmill exercise (RATE)	7	63 ± 13		RATE with usual care, including physical, occupational, and speech and language therapy	Not specified	30–60 min/day; 4–5 times/week	
Faulkner et al., 2013_A	Exercise and education program	30	68 ± 11	BP, TC, HDL, FBG	Aerobic exercise (treadmill, cycle ergometer), resistance exercise, core stability, balance, control, postural exercises, flexibility, etc., and focused education on prevention	50%–85% HRR	90 min; 2 times/week	• Exercise combined with an education program significantly improves cardiovascular fitness and reduces the risks of post-stroke complications, this improvement can be maintained 3 months post-intervention
Faulkner et al., 2013_B	Usual care	30	69 ± 10		Standard care	Not specified	Not specified	
Kirk et al., 2013_A	Cardiac rehabilitation	12	67.5 ± 11.4	BP, TC, HDL, FBG	Aerobic, anaerobic exercise with usual care and education	50%–70% HR_max_	In three phases; 1 h in each phase	• Standard cardiac rehabilitation programs are feasible and effective in reducing the risk of future cardiovascular events for patients after minor and transient ischemic stroke
Kirk et al., 2013_B	Usual care	12	66.8 ± 7.3		Usual care	Not specified		
Kono et al., 2013_A	Lifestyle modification and education	35	63.5 ± 7.0		Walking at home, reducing salt intake, health education, center-based aerobic and resistance training	60%–70% HRR	60 min, 3–5 times/week	• Lifestyle modification is beneficial for reducing stroke recurrence and improving SBP and HDL levels
Kono et al., 2013_B	Usual care	35	63.4 ± 11.4		Health education and usual care	Not specified	Not specified	
Lennon et al._A	Active	24	59.0 ± 10.3		Cycle ergometry with resistance, stress management education and usual care	60% of HRR	30 min	• Cardiac rehabilitation programs improve fitness and reduce complications after an acute ischemic stroke event
Lennon et al._B	Control	24	60.5 ± 10.0		Only physical and occupational therapy	Not estimated	Not estimated	
MacKay-Lyons et al., 2022_A	Prevention	94	64.3 ± 10.2		Health education, aerobic and strength training	60%–80%	60 min, 5 times/week	• This study concluded that patients with exercise and preventive education significantly improved LDL and DBP, but these improvements were not sustained after a few months
MacKay-Lyons et al., 2022_B	Usual care	90	65.9 ± 9.9		Usual care	Not estimated	Not estimated	

BP, blood pressure; cSBP, central systolic blood pressure; HR, heart rate; HRR, heart rate reserve; CO, cardiac output; CBF, cerebral blood flow; TC, total cholesterol; LDL, low-density lipoprotein; HDL, high-density lipoprotein; FVC, forced vital capacity; FEV, forced expiratory volume; PEF, peak expiratory flow; 6MWT, 6 min walking test; 10MWT, 10 min walking test; TUG, timed up and go; HI, high-intensity; LI, low-intensity; RPE, ratings of perceived exertion; Aix, augmentation index; QoL, quality of life; FBG, fasting blood glucose; VO_2peak_, peak volume of oxygen.

### 2.5 Statistical analysis

We analyzed baseline and post-intervention effect data from included studies’ hemodynamic changes (BP, CO, HR), lipid profile (HDL, LDL, TC, TG), FBG, functional capacity (6MWT, TUG, BBS), and exercise capacity by VO_2peak_. We considered each rehabilitation program from a single study as a distinct entry for analysis; we validated the final results of each variable with changes at post-intervention of each study's control and intervention group illustrated by the study's author, followed by the published methodology (34). We used the random effects model of meta-analysis, converted standard error data to standard deviation, and estimated the mean difference (MD) with a 95% confidence interval (CI). A *P-*value of <0.05 was considered statistically significant. We utilized the software “RevMan” (35) version 5.3 and *STATA* version 17.0 (StataCorp, College Station, TX, United States) for data analysis. We assessed heterogeneity by the *I*^2^ value (inconsiderable heterogeneity, *I*^2^ < 0%–30%) and the funnel plot to identify potential outliers (36).

## 3 Results

### 3.1 Study selection and screening

**Figure 1:**
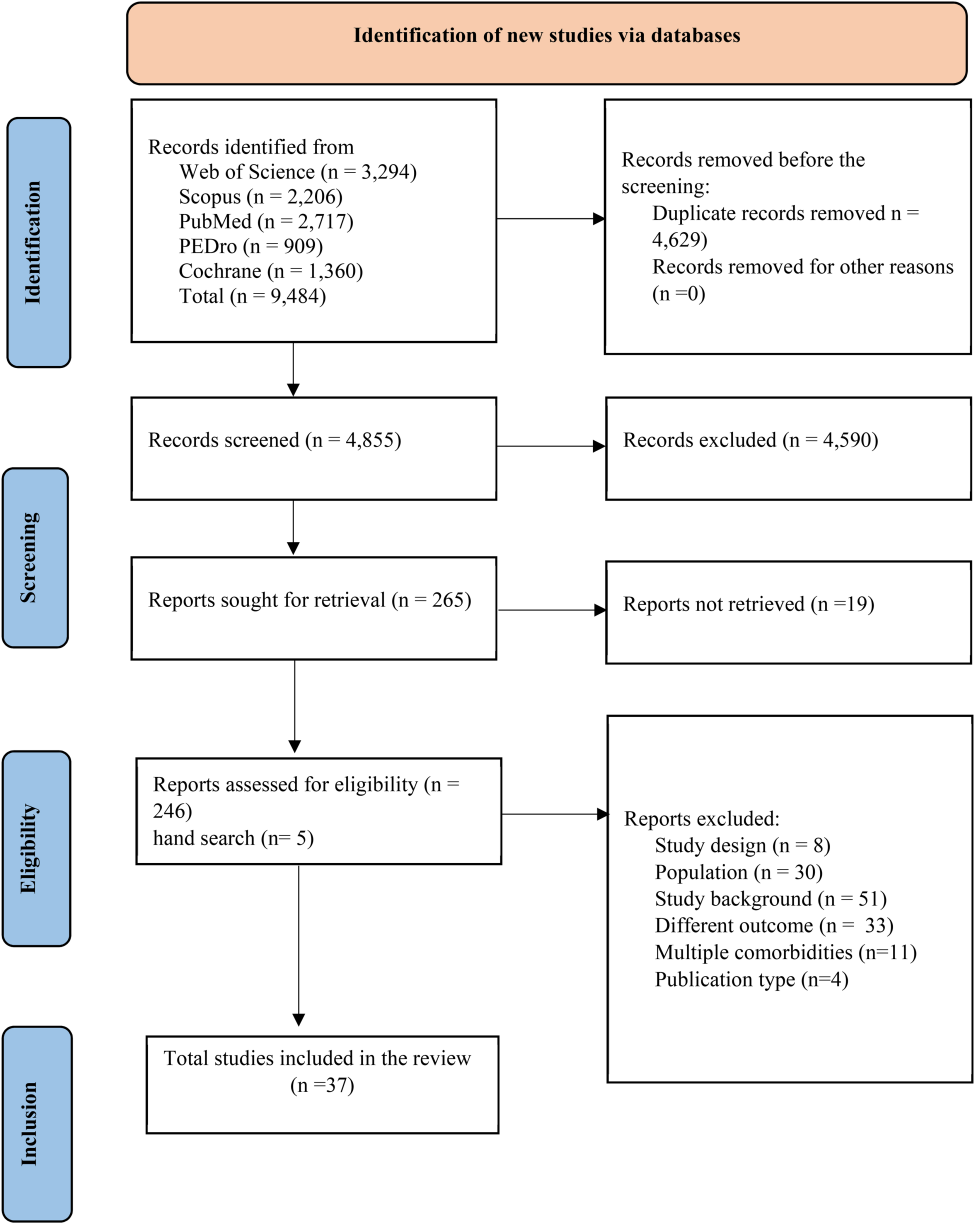
Study selection process from online databases (PRISMA guideline).

A total of 9,484 articles were retrieved, and all duplicate articles were removed. A thorough screening of titles and abstracts excluded 4,855 articles, and 265 articles were assessed for eligibility through full-text analysis. Finally, 37 articles satisfied all the inclusion criteria. The PRISMA flow diagram illustrates the overall search strategy (Figure 1), and the findings of all keywords from electronic databases were tabulated (Supplementary File 1). PRISMA checklist for the abstract, full text, and other information are available in Supplementary File 2.

### 3.2 Study characteristics

The characteristics and rehabilitation programs of the included articles were compiled in Tables 1 and 2. The data from 37 trials (37–73), all intervention and control groups, were illustrated separately in Table 2. In total, 2,337 stroke patients [minimum age of (mean ± SD) 54 ± 8.98 years and a maximum of (mean ± SD) 74.7 ± 9.3 years] who participated in various rehabilitation programs (minimum duration of 4 weeks and maximum of 24 weeks). The inclusion criteria among articles on stroke incidence among included participants was a minimum of one week to a maximum of a year post-stroke. Ischemic stroke cases comprised 52.11% (*n* = 1,218) of all stroke events (Table 1). Moore et al. used the same subjects but reported different variables in two (45, 46) studies in different periods.

Additionally, sixteen articles (37, 38, 40, 41, 43, 44, 49, 53, 55, 57, 63–65, 67, 69, 72) investigated aerobic exercise (such as walking and cycling); six articles (40, 45, 48, 54, 59, 62) compared the effect of health education training; one article (50) included exergaming exercises; one article (70) involved robot-assisted walking training; and three articles (39, 42, 73) involved dynamic and resistance training. Exercise sessions lasted no more than 60 min, were performed three times/week, and were of varying intensity based on ratings of perceived exertion (14–16) and maximum heart rate (40%–95%). Exercise-based rehabilitation programs of all included articles are tabulated in Table 2.

### 3.3 Risk of bias and study quality

All included articles were evaluated using the “PEDro” and the ROB 2.0 tool. According to the PEDro score, 26 articles (37–41, 45, 46, 48–50, 53–56, 58, 59, 61, 62, 65, 66, 68–73) scored between 6 and 8, which is considered significantly good quality, and the remaining articles were regarded as fair quality (Table 1). None of the articles was excluded due to low quality. Furthermore, only three articles (39, 55, 57) had a high risk, and nineteen articles (42, 46, 48, 49, 53–56, 58, 59, 61, 63, 65, 67–71, 73) had a low risk of bias according to the results of the ROB 2.0 tool. Some concerns were noted in other articles due to the selection of reported results and the randomization process (Supplementary File 3).

### 3.4 Post-rehabilitation changes in BP, HR, and CO

**Figure 2:**
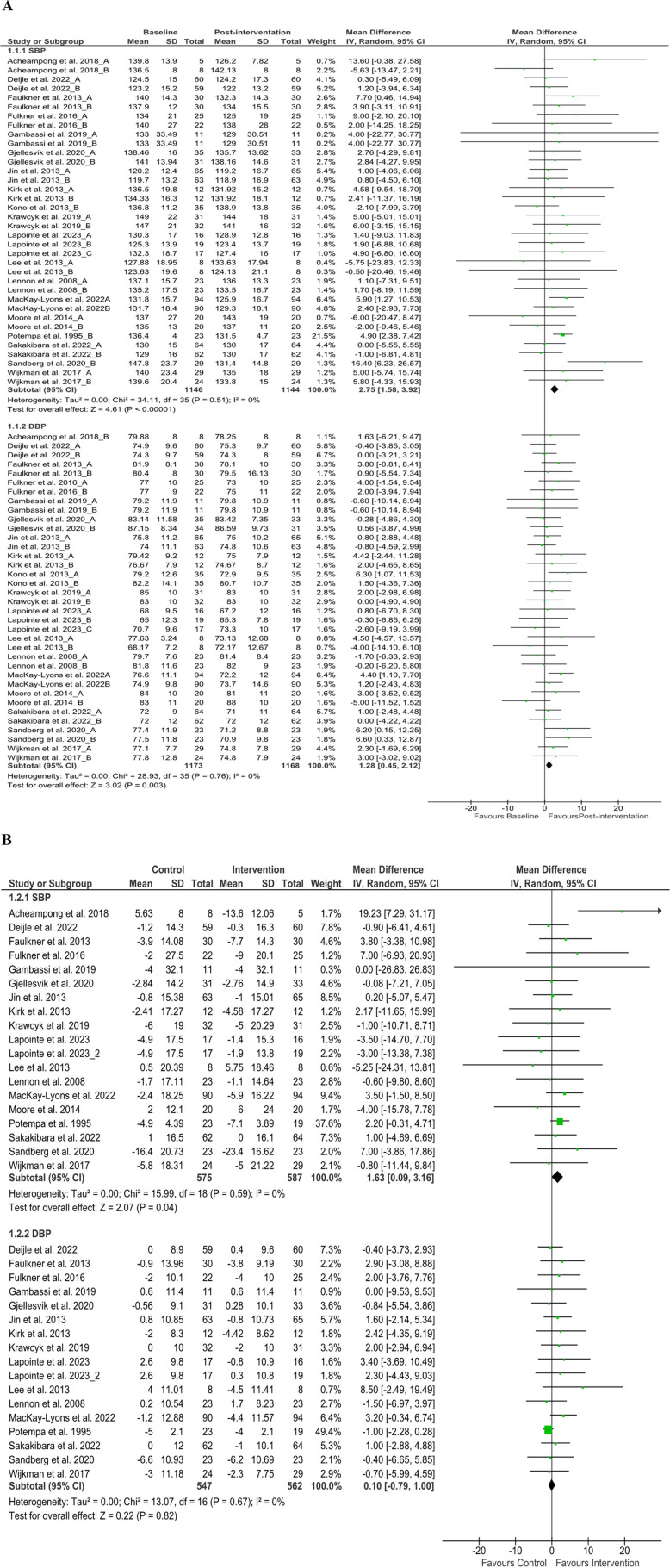
Blood pressure changes **(A)** baseline to post-intervention and **(B)** difference in pre- and post-intervention at the control and intervention groups after post-stroke rehabilitation programs. SBP, systolic blood pressure; DBP, diastolic blood pressure; SD, standard deviation; IV, inverse variance; CI, confidence interval; df, degree of freedom.

**Figure 3:**
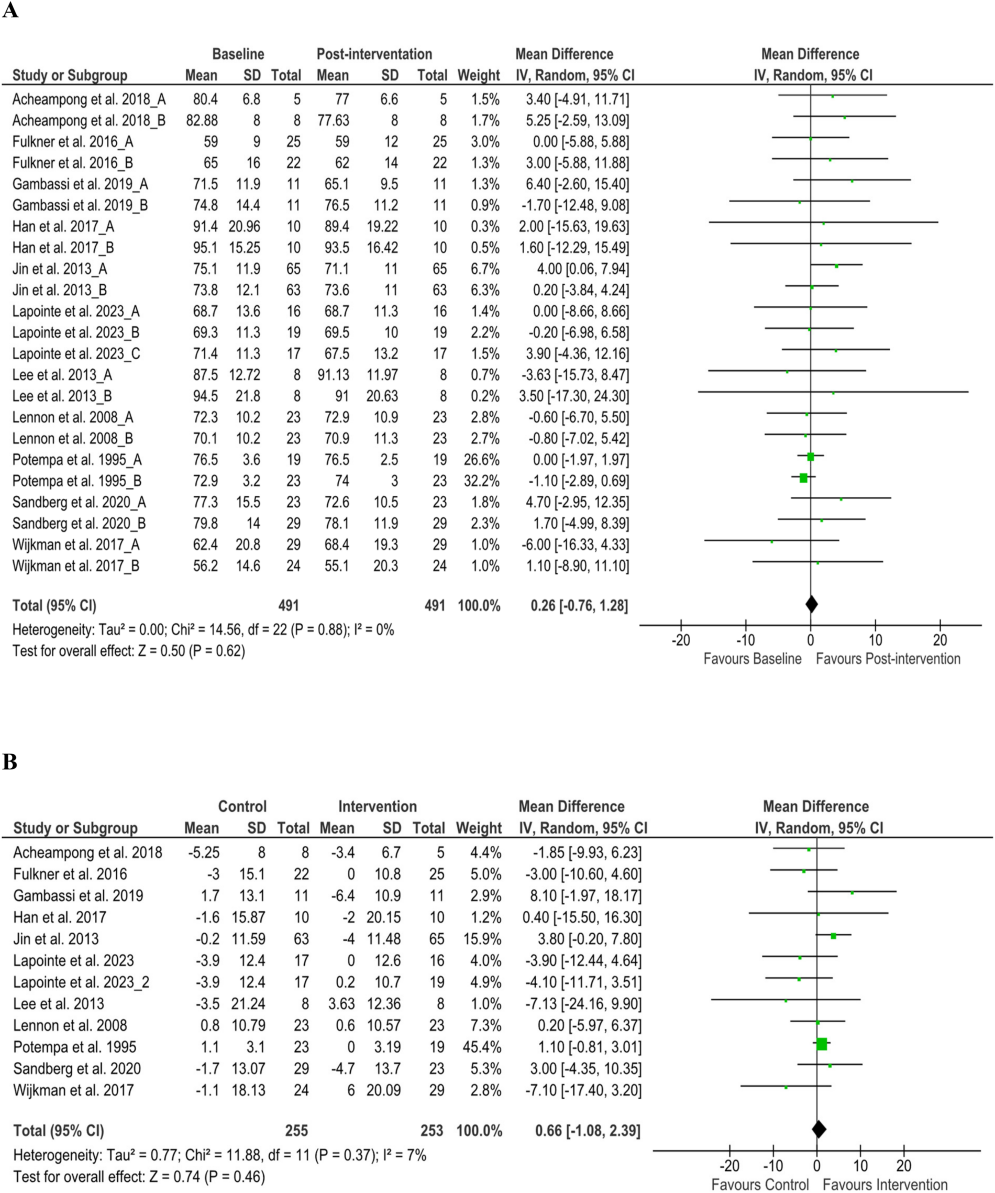
Heart rate changes **(A)** baseline to post-intervention and **(B)** difference in pre- and post-intervention at the control and intervention groups after post-stroke rehabilitation programs. SD, standard deviation; IV, inverse variance; CI, confidence interval; df, degree of freedom.

From 19 articles (37–40, 44, 45, 48, 52, 54, 57–62, 64, 68, 69, 72), we analyzed stroke patients’ SBP from baseline (number of patients, *n* = 1,146) and after discharge (*n* = 1,144). Cumulative results showed that the reduction of SBP after discharge was significant (MD 2.75 mmHg; 95% CI 1.58–3.92; *P* < 0.05, *I*^2^ = 0%), similar results were found in the comparison of baseline and discharge changes between control and intervention group (*P* < 0.05) (Figure 2), but the subgroup analysis of aerobic exercise, resistance training, and standard care from baseline to discharge shows insignificant reduction but tends to be positive effect (Supplementary File 4A). Notably, diastolic blood pressure (DBP) from baseline (*n* = 1,173) and after discharge (*n* = 1,168) from 18 articles (37–40, 44, 45, 48, 52, 54, 57–62, 68, 69, 72) showed significant declination (MD 1.28 mmHg; 95% CI 0.45–2.12 mmHg; *P* < 0.05, *I*^2^ = 0%), but in the comparison of baseline and discharge changes between control and intervention group, this improvement was insignificant (*P* > 0.05, *I*^2^ = 0%) as well as from all subgroup analysis (Supplementary File 4B). We analyzed stroke patients’ physiological variables (such as HR and CO) to find insightful explanations for these challenges. We found post-rehabilitation HR changes from eleven articles (38, 39, 44, 52, 56, 57, 60, 61, 64, 68, 72) (Figure 3) and CO changes from three articles (41, 45, 46) (Supplementary File 5) at discharge, and the comparison of baseline and discharge changes between the control and intervention group was insignificant (*P* > 0.05) but ameliorative. However, concerning the positive impact of rehabilitation, our study recommends modification of post-stroke rehabilitation protocol in terms of exercise intensity, duration, and frequency to have a significant outcome.

### 3.5 Post-rehabilitation changes in lipid profile

**Figure 4:**
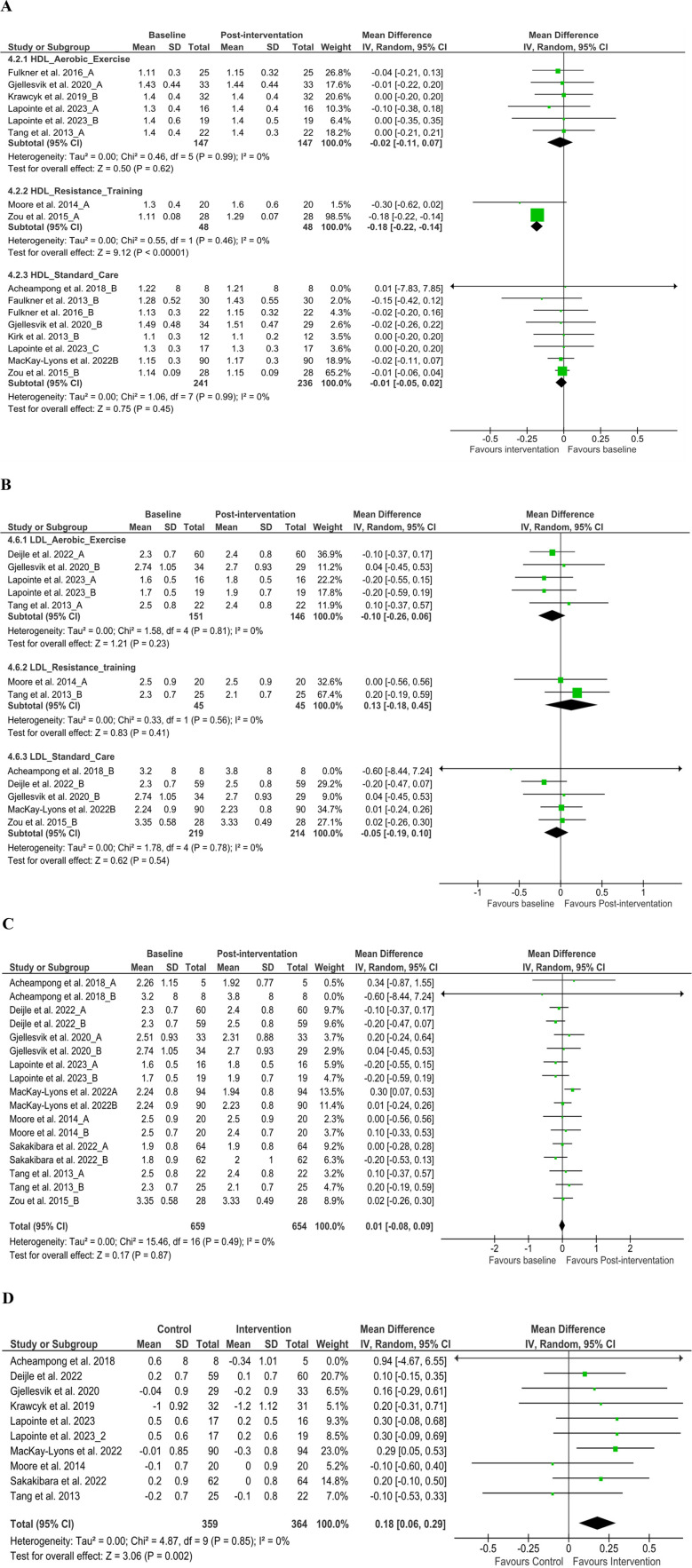
Subgroup analysis from baseline to post-intervention changes. **(A)** High-density lipoprotein and **(B)** low-density lipoprotein, **(C)** low-density lipoprotein difference on baseline and post-intervention, **(D)** low-density lipoprotein changes in the control and intervention groups after post-stroke rehabilitation programs. SD, standard deviation; IV, inverse variance; CI, confidence interval; df, degree of freedom.

We analyzed HDL from 12 articles (38, 40, 44, 45, 48, 49, 52, 54, 58, 62, 69, 73) (*n* = 750) at discharge [MD −0.02 (95% CI −0.05 to 0.01), *P* > 0.05, *I*^2^ = 0%], and the comparison of baseline and discharge changes between the control and intervention group [MD −0.04 (95% CI −0.10 to 0.02), *P* > 0.05, *I*^2^ = 0%] was insignificant (Supplementary File 6A). However, subgroup analysis at discharge on resistance training found significant changes [MD −0.18 (95% CI −0.22 to 0.14), *P* < 0.05, *I*^2^ = 0%] (Figure 4A). LDL from nine articles (37, 40, 44, 45, 48, 49, 52, 62, 73) (*n* = 659) at discharge [MD 0.01 (95% CI −0.08 to 0.09), *P* > 0.05, *I*^2^ = 0%] and subgroup analysis at discharge was insignificant (Figures 4B,C). Further, the comparison of baseline and discharge changes between the control and intervention group found significant changes [MD 0.18 (95% CI 0.06–0.29), *P* < 0.05, *I*^2^ = 0%] (Figure 4D). These changes suggest that all intervention groups’ exercise may have had a higher impact due to the type of exercise combination than those of control groups at discharge, which requires further validation using cross-over control trial methods. TC from 10 articles (37, 38, 40, 45, 49, 52, 54, 58, 61, 73) (*n* = 491) found insignificant improvement at discharge and between groups (*P* > 0.05) (Supplementary File 7). Nonetheless, TG from six articles (37, 49, 52, 62, 69, 73) (*n* = 399) found significant improvement at discharge [MD 0.10 (95% CI 0.01–0.18), *P* < 0.05, *I*^2^ = 0%] (Supplementary File 8A). Furthermore, the comparison of baseline and discharge changes between the control and intervention group found insignificant changes [MD 0.10 (95% CI −0.04 to 0.24), *P* < 0.05, *I*^2^ = 0%] (Supplementary File 8B).

### 3.6 Exercise and functional capacity after rehabilitation

**Figure 5:**
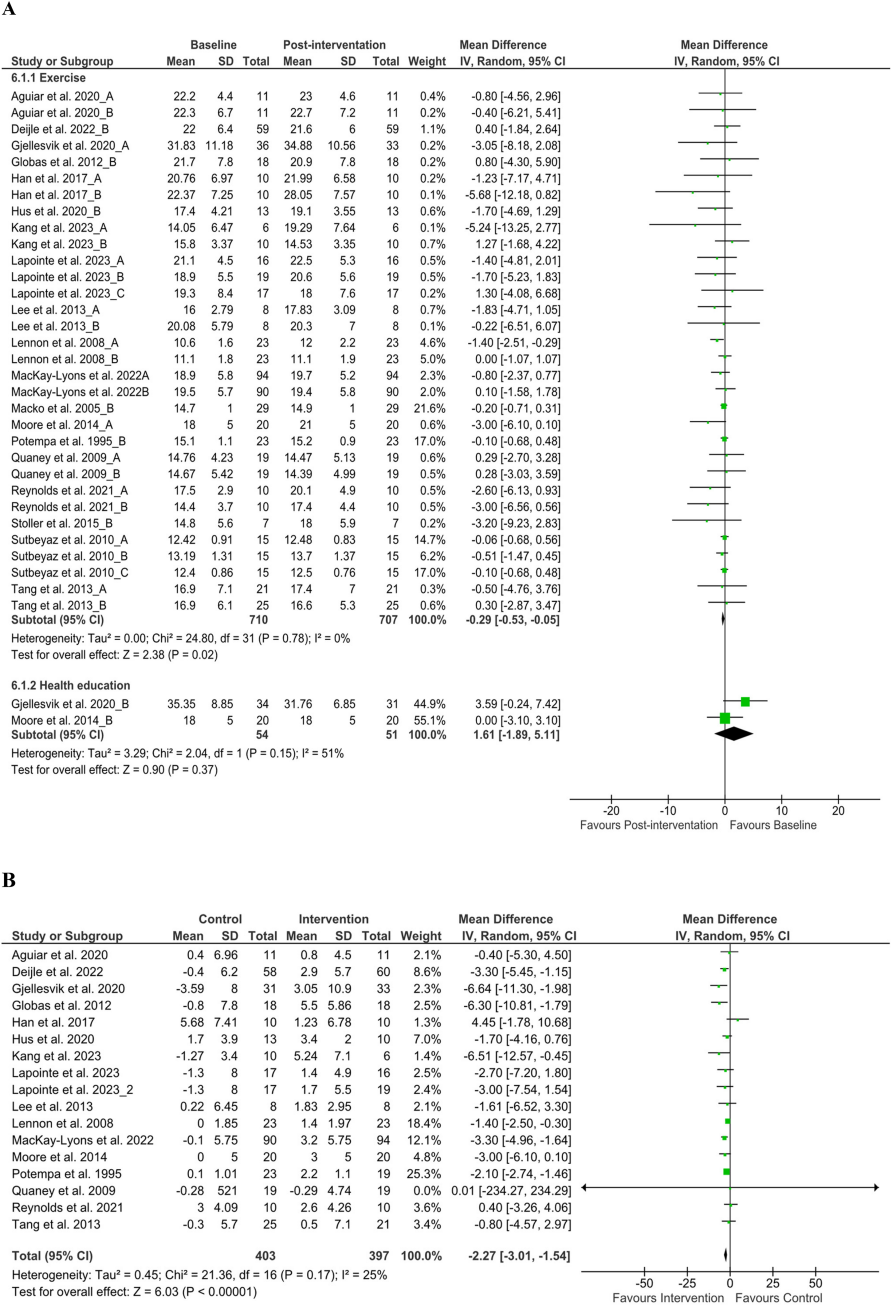
**(A)** VO_2_ changes baseline to post-intervention and **(B)** difference in pre- and post-intervention at the control and intervention groups. SD, standard deviation; IV, inverse variance; CI, confidence interval; df, degree of freedom.

Post-rehabilitation exercise capacity was assessed via VO_2peak_ after exercise from nineteen articles (37, 40–42, 44, 45, 49, 53, 55, 56, 60–66, 70, 71) (*n* = 710) and found significant changes at discharge [MD −0.29 ml/kg/min (95% CI −0.53 to −0.05), *P* < 0.05, *I*^2^ = 0%], although insignificant changes only after health education (*P* > 0.05), but the inclusion of health education with standard care and exercise-based rehabilitation was found to have a positive effect. However, a significant improvement was found in the comparison of baseline and discharge changes between the control and intervention group [MD −2.27 ml/kg/min (95% CI −3.01 to −1.54), *P* < 0.05, *I*^2^ = 0%] (Figure 5).

Post-stroke rehabilitation significantly improved functional capacity measured in 6MWT from 12 articles (42, 43, 45, 49, 50, 53, 55–57, 60, 63, 66) (*n* = 448) and found significant changes at discharge [MD −27.15 m (95% CI −45.11 to −9.18), *P* < 0.05, *I*^2^ = 49%], but the comparison of baseline and discharge changes between the control and intervention group found insignificant changes [MD −13.61 m (95% CI −39.95 to 12.73), *P* < 0.05, *I*^2^ = 31%] (Supplementary File 9). Furthermore, BBS (*n* = 123) improved significantly at discharge than baseline [MD −3.39 (95% CI −5.04 to −1.75), *P* < 0.05, *I*^2^ = 52%], as well as changes between control and intervention groups from one article (45) (*n* = 40) (*P* < 0.05) (Supplementary File 10). Contrarily, TUG (39, 46) (*n* = 62) test has an insignificant change after post-stroke rehabilitation at discharge [MD 1.76 (−0.49 to 4.01), *P* > 0.05, *I*^2^ = 0%] and the comparison of baseline and discharge changes between the control and intervention groups [MD 2.67 (−0.81 to 6.14), *P* > 0.05, *I*^2^ = 0%] (Supplementary File 11). These results suggest an overall deterioration in functional outcomes in different measures. In line with previous studies, our study also emphasizes that standardized and personalized measurement tools must be developed to prescribe exercise for people with stroke, concerning exercise principles such as specificity, overload, and reversibility for better outcomes (74).

Furthermore, FBG from seven articles (38, 48, 49, 54, 58, 62, 73) (*n* = 544) found significant changes at discharge [MD 0.15 (95% CI 0.04–0.26), *P* < 0.05, *I*^2^ = 0%] and the comparison of baseline and discharge changes between the control and intervention group [MD 0.17 (95% CI 0.03–0.30), *P* < 0.05, *I*^2^ = 0%] (Supplementary File 12). Moreover, homocysteine level changes from two articles (48, 49) (*n* = 87) found an insignificant (*P* > 0.05) improvement after the post-stroke rehabilitation program (Supplementary File 13).

### 3.7 Publication bias and sensitivity analysis

We analyzed publication bias (75) using the funnel plot for variables, which included over ten studies; none of our results presented potential bias (shown in Supplementary Files 4, 6, 8, 10, 11). Sensitivity analysis (76) was done using the leave-one-out method. If any studies significantly impact overall results on any variables, we excluded that study from the analysis. Moreover, our findings suggest that all post-rehabilitation interventions enact no potential risk on outcomes.

## 4 Discussion

**Figure 6:**
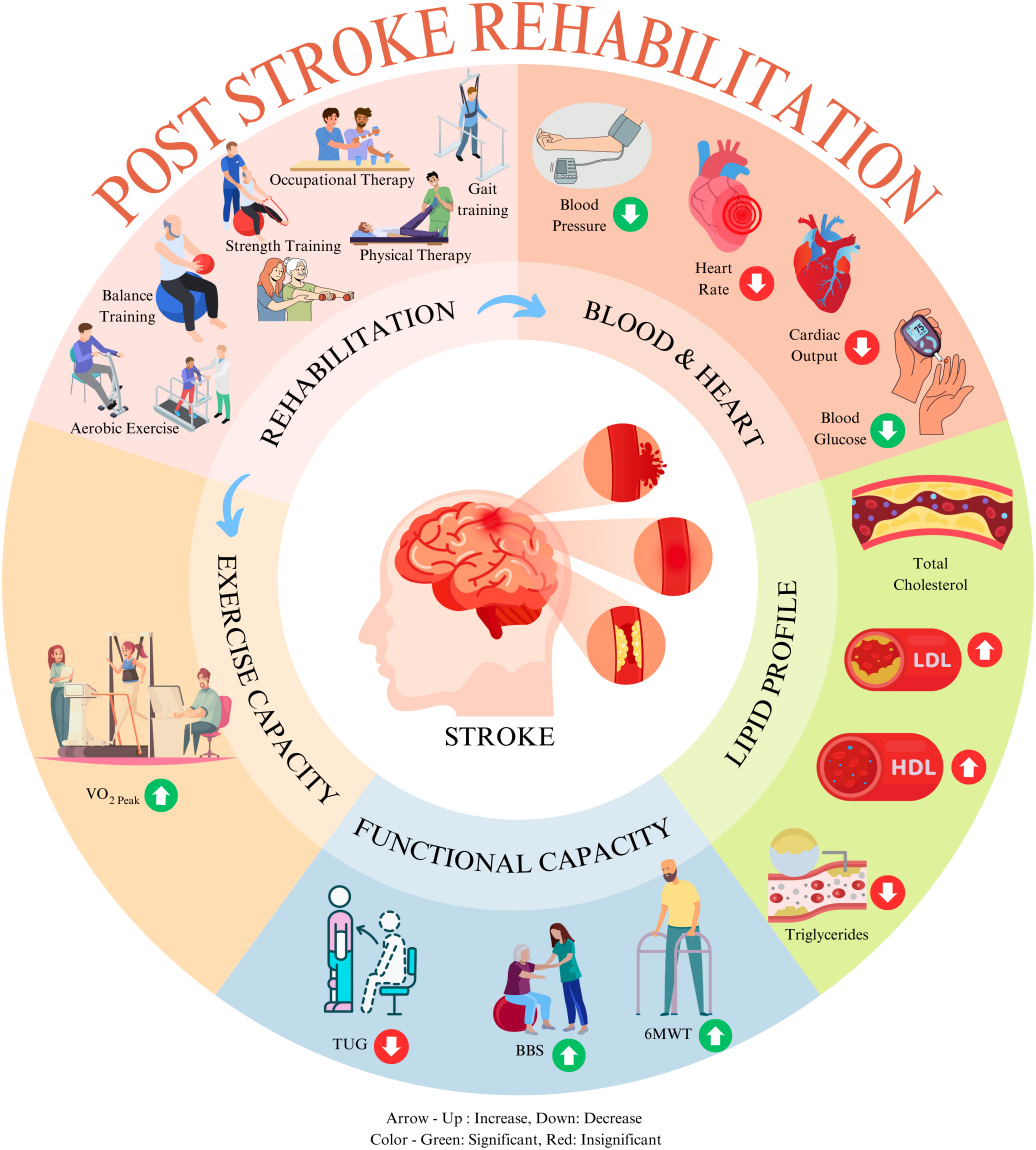
Effect of exercise-based rehabilitation among post-stroke patients at discharge. This image was created using elements from Canva, licensed under Free Content License.

This systematic review and meta-analysis sought to evaluate the extent to which a rehabilitation program impacts cardiac health (BP, HR, and CO), lipid profile variables (HDL and LDL), exercise capacity (VO_2peak_), and functional capacity (6MWT) in patients after stroke. We included all RCTs that evaluated these changes among stroke survivors at any stage. Using the meta-analysis method, we analyzed the outcome data for the mean difference at discharge from baseline from all groups and changes (baseline to post-intervention) at discharge between control and intervention groups. The results of all variables at discharge are graphically presented in Figure 6. This result provides a comprehensive conclusion on the overall exercise-based rehabilitation programs practiced for patients with stroke. Notably, our study indicated that, whereas BP, functional, and exercise capacity improved significantly following rehabilitation programs, lipid level control was insignificant but ameliorative. These findings support modifying the post-stroke rehabilitation protocol and prioritizing cardiac health as a surrogate measure of rehabilitation outcome.

BP reduction is vital to controlling stroke risk factors (26). Numerous pieces of evidence stated that >5.2 mmHg reduction of SBP can reduce the odds of having a recurrent stroke by up to 22% (77). Among combined exercise training groups, SBP and DBP reduction was significant. However, subgroup analysis showed an inconsistent effect, supporting both findings from a Cochrane review among 2,797 patients and a meta-analysis, which compared only aerobic exercise effects after rehabilitation and found an inconsistent effect on SBP and DBP (77, 78). Our analysis indicates that the underlying reason for this inconsistency could be the effect of exercise intensity. A recent RCT study emphasized that the intensity of training programs during stroke rehabilitation is pivotal to improving cardiac health and functional capacity (79). High-intensity treadmill training at a peak heart rate of 85%–95% (40) and high-intensity aerobic exercise training (brisk walking, cycling, marching) among 50 stroke patients showed significantly improved functional capacity (49). Nevertheless, growing evidence suggests that exercise intensity and exercise-induced fatigue burden patient recovery during rehabilitation (20).

HR is a precursory variable for assessing and reducing cardiovascular risk factors (80). After ischemic stroke, a higher HR at baseline correlated to higher cardiovascular risk and mortality (81). Mean HR increased to 10 beats/minute (bpm) from baseline (60 bpm), increasing the cardiovascular risk hazard ratio to approximately 0.39 (82, 83). Thirty-day mortality increases by 2.5% in ischemic stroke patients with atrial fibrillation for mean HR increases each one bpm over 80 bpm (81). HR and HRV changes occur inversely (84). Nozoe et al. illustrated that early mobilization after an ischemic stroke would cause neurological deterioration, which diverges the sympathetic nervous activity to affect HRV, identified by the fraction of low frequency and high frequency (19). In clinical practice, to identify and adjust the HRV to find the best possible training program for a stroke patient, a new training method called “the self-generate physiological coherence system” was designed based on the brain–heart interaction and pressure concept, demonstrating higher recovery and patient satisfaction (20). Our findings, backed by other studies, found that after a rehabilitation program, the resting HR of stroke patients decreased insignificantly (18, 80). These findings suggest that to develop a personalized rehabilitation program, one needs to focus on HRV and plan to decrease resting HR.

Furthermore, increased HDL reduces the risk of ischemic stroke (85). Conversely, an LDL level of <3.9 mmol/L after stroke can minimize cardiovascular risk (86). Our study is in line with previous findings that the reduction of LDL and TC and improvement of HDL are insignificant after post-stroke rehabilitation (25, 77). However, we found that after resistance exercise, HDL improvement was significant. Yang and colleagues (87) found a robust correlation between a decrease in total cholesterol/HDL ratio and an increase in VO_2peak_, although the level of evidence was reported as low. Our meta-analysis of four RCTs on post-stroke rehabilitation reported an improvement in VO_2peak_ in comparing baseline and discharge changes between the control and intervention group in MD −2.97 ml/kg/min (95% CI −3.01 to −1.54). This finding is similar to two other meta-analyses, 1 of 13 RCTs in MD 2.53 ml/kg/min (95% CI 1.78–3.29) and another of 12 RCTs in MD 2.27 ml/kg/min (95% CI 1.58–2.95) on cardiorespiratory fitness in stroke patients’ after exercise (88, 89). Therefore, 1 ml/kg/min of VO_2peak_ improvement reduces 15% of mortality risk among coronary artery patients (90). However, mortality risk after stroke increases by elevated HR rather than the level of VO_2peak_ of patients with stroke (81). Furthermore, a Cochrane review stated that cardiorespiratory fitness training is feasible for the stroke population and improves walking capability and balance (78). Our findings also showed that stroke survivors covered significantly greater walking distances in 6MWT and BBS scores improved after rehabilitation.

Improving health-related knowledge among stroke patients can also improve their cardiac health (91). One of our included studies used an Android health application among 1,299 stroke patients to remind them about a healthy lifestyle through voice and text message services. Significant improvements in their cardiac health, such as BP and lipid profile, were found (51). A nurse-led health education study, including 268 patients, showed similar findings (47). Furthermore, In line with previous studies, specific exercise-based rehabilitation (aerobic, resistance) can sufficiently improve post-stroke blood pressure and functional or exercise capacity; yet, the improvement on some cardiac variables (HR, CO) or lipid profile variables (LDL, TC) is still inconclusive (55, 57, 64, 73). A growing number of RCT studies compared the effects of exercise-based rehabilitation with sham groups, while the intervention group exhibits a higher impact due to program design (55, 65, 72). To alleviate these, we recommend more cross-over randomized control trials on our study variables among post-stroke patients. However, answering the root cause of this decline is beyond our study objectives; more fundamental studies on the mechanism of NSC are recommended, as mentioned before. Thus, our study, in line with other meta-analyses, suggests that aerobic exercise has higher benefits than other exercise training and should be included as a fundamental exercise program for stroke survivors (23, 25).

Moreover, Stoller and colleagues (70) experimented with robot-assisted training and illustrated that the recommended intensity is not consistently achievable among stroke patients. Increasing exercise repetition might positively impact stroke patients’ exercise outcomes (92), which requires robust evidence from clinical studies. Some studies mentioned that the HRR was at a high-intensity level (70%–85%); the evidence is still disseminated to determine the optimal exercise intensity level for stroke patients (54–56, 62). Nonetheless, during follow-up, the impact of exercise was found to have a deterioration than at the discharge level (54, 62), which may hinder overall health among stroke survivors; practicing health education (40, 59, 62) and home-based (38, 48, 69) and community-based (42) exercise programs might be beneficial and improve post-stroke QoL and mortality.

Eventually, we recommend further studies in a large cohort in a randomized and cross-over control trial setting using modern technology such as a smartwatch and functional near-infrared spectroscopy to compare exercise with different intensities and repetition with health education with long-term follow-up to find the rehabilitation effects on cardiac health. A meta-analysis is required to find different exercises that impact blood pressure changes and report the risk of fatigue, syncope, and mortality rate.

### 4.1 Limitation

Post-stroke rehabilitation intensely focused on the functional outcome rather than cardiac health, which led to the inclusion of fewer articles on our study topic. Our study selection criteria were not limited to treatment methods, intensity, or the stroke timeline, which generalizes our findings on rehabilitation practice. Due to data unavailability, we could not include all studies in all variables during the meta-analysis; a subgroup analysis on the time of stroke incidence and the impact of exercise was also unattainable. Considering the significance of hemodynamic changes among this population, we suggest that future research on the effect of post-stroke rehabilitation should report changes in the hemodynamic variables as reciprocal measures.

## 5 Conclusion

Our study revealed that current exercise-based rehabilitation programs significantly improve blood pressure and exercise capacity in patients with stroke at discharge. However, lipoprotein changes remained inconclusive. Although ameliorative changes were noted in most variables, more research is needed to determine optimum exercise intensity, type combination, and health education to reduce post-stroke complications and mortality.

## Data Availability

The original contributions presented in the study are included in the article/Supplementary Material, further inquiries can be directed to the corresponding author.
