# Supplementary material for: Current exercise-based rehabilitation impacts on poststroke exercise capacity, blood pressure, and lipid control: a meta-analysis
Source: Front Cardiovasc Med. 2025 Mar 24;12:1457899. doi: 10.3389/fcvm.2025.1457899 (PMC11973393; doi:10.3389/fcvm.2025.1457899)

Supplementary Material

1. Supplementary file 1: Search strategy of study selection.
2. Supplementary file 2: PRISMA checklist for abstract and full text.
3. Supplementary file 3: Risk of bias among included articles.
4. Supplementary file 4: Sub-group analysis from baseline to post-intervention changes (A) systolic blood pressure, (B) diastolic blood pressure after rehabilitation, (C) Funnel Plot.
5. Supplementary file 5: Cardiac output changes (A) baseline to post-intervention, (B) difference in pre-and post-intervention at control and intervention groups after rehabilitation.
6. Supplementary file 6: (A) High-density lipoprotein changes from baseline to post-intervention, (B) difference in control and intervention groups, (C) HDL Funnel Plot, (D) LDL Funnel Plot.
7. Supplementary file 7: Total cholesterol changes (A) baseline to post-intervention, (B) difference in pre-and post-intervention at control and intervention groups after rehabilitation, (C) Funnel Plot.
8. Supplementary file 8: Triglycerides changes (A) baseline to post-intervention, (B) difference on pre-and post-intervention at control and intervention groups after rehabilitation, (C) Funnel Plot.
9. Supplementary file 9: 6MWT changes (A) baseline to post-intervention, and (B) difference in pre-and post-intervention at control and intervention groups after poststroke rehabilitation programs, (C) Funnel Plot.
10. Supplementary file 10: Berg Balance score changes (A) baseline to post-intervention, and (B) difference in pre-and post-intervention at control and intervention groups after poststroke rehabilitation programs. (SD: Standard deviation; IV: Inverse-variance, CI: Confidence interval; df: degree of freedom)
11. Supplementary file 11: Time-up and go score changes (A) baseline to post-intervention and (B) difference in pre-and post-intervention at control and intervention groups after poststroke rehabilitation programs.
12. Supplementary file 12: Fasting blood glucose level changes (A) baseline to post-intervention, (B) difference in pre-and post-intervention at control and intervention groups after rehabilitation, (C) Funnel Plot.
13. Supplementary file 13: Homocysteine level changes (A) baseline to post-intervention, and (B) difference in pre-and post-intervention at control and intervention groups after poststroke rehabilitation programs.

Supplementary file 1: Search strategy of study selection**.**

Search No	Search Term	Database, Advanced Search Mode, and Limiters	Reasons	Number of articles (June 2024)	
#1	Stroke, Cerebr*, Brain	Web of Science: Topic, Boolean/Phrase: OR	To identify the related articles on the study subject.	4,351,274	
		Scopus: TITLE-ABS-KEY, Boolean/Phrase: OR		3,414,054	
		PubMed: All fields, Boolean/Phrase: OR		3,007,630	
#2	Ischemic, hemorrhagic, embolic, thrombotic	Web of Science: Topic Boolean/Phrase: OR	To find out articles on disease type.	732,678	
		Scopus: TITLE-ABS-KEY, Boolean/Phrase: OR.		695,979	
		PubMed: All fields, Boolean/Phrase: OR		6,258,055	
#3	Cardi*, heart	Web of Science: Topic Boolean/Phrase: OR, Terms:	To identify articles on study variables	6,347,154	
		Scopus: TITLE-ABS-KEY, Boolean/Phrase: OR		3,882,982	
		PubMed: All fields, Boolean/Phrase: OR		3,206,646	
#4	rehab*, exercise, training	Web of Science: Topic, Boolean/Phrase: AND	To find out articles on study intervention.	4,666,895	
		Scopus: TITLE-ABS-KEY, Boolean/Phrase: AND		2,788,012	
		PubMed: All fields, Boolean/Phrase: AND		3,589,718	
#5	#1 AND #2	Web of Science: Topic, Boolean/Phrase: AND	To combine articles on the subject with different disease types.	310,369	
		Scopus: TITLE-ABS-KEY, Boolean/Phrase: AND		274,856	
		PubMed: All fields, Boolean/Phrase: AND		835,494	
#6	#3 AND #4	Web of Science: Topic, Boolean/Phrase: AND	To combine articles on different variables and interventions.	464,219	
		Scopus: TITLE-ABS-KEY, Boolean/Phrase: AND		296,634	
		PubMed: All fields, Boolean/Phrase: AND		376,434	
#7	#5 AND #6	Web of Science: Topic, Boolean/Phrase: AND, Open access, Clinical trial, 2012 to 2022.	To isolate articles according to PICO(S).	3,294	
		Scopus: TITLE-ABS-KEY, Boolean/Phrase: AND, Open access, journal article, 2012 to 2022		2,206	
		PubMed: All fields, Boolean/Phrase: AND, Open access, Randomized control trial, 2012 to 2022.		2,717	
PEDro		Article & abstract: Stroke Exercise "blood pressure" Method: Clinical Trial Bolen Phase: AND		58	909
		Article & abstract: Stroke “heart rate” Method: Clinical Trial Bolen Phase: AND		73	
		Article & abstract: Stroke Exercise lipid Method: Clinical Trial Bolen Phase: AND		13	
		Article & abstract: Stroke Exercise Method: Clinical Trial Bolen Phase: AND		765	

Cochrane Library	Title, abstract, Keyword: (Stroke OR Cerebr* OR Brain) AND (Ischemic OR hemorrhagic OR embolic OR thrombotic) AND (Exercise OR Training OR Education) Source: Trials, Embase	1360

Supplementary file 2: PRISMA checklist for abstract and full text.

Section and Topic	Item #	Checklist item	Location where item is reported
**TITLE**			
Title	1	Identify the report as a systematic review.	Title page
**ABSTRACT**			
Abstract	2	See the PRISMA 2020 for Abstracts checklist.	Title and abstract page
**INTRODUCTION**			
Rationale	3	Describe the rationale for the review in the context of existing knowledge.	2nd paragraph
Objectives	4	Provide an explicit statement of the objective(s) or question(s) the review addresses.	3rd paragraph
**METHODS**			
Eligibility criteria	5	Specify the inclusion and exclusion criteria for the review and how studies were grouped for the syntheses.	3rd Paragraph
Information sources	6	Specify all databases, registers, websites, organisations, reference lists and other sources searched or consulted to identify studies. Specify the date when each source was last searched or consulted.	last paragraph
Search strategy	7	Present the full search strategies for all databases, registers and websites, including any filters and limits used.	Flowchart 1 and in the supplemental file 1
Selection process	8	Specify the methods used to decide whether a study met the inclusion criteria of the review, including how many reviewers screened each record and each report retrieved, whether they worked independently, and if applicable, details of automation tools used in the process.	selection of studies paragraph
Data collection process	9	Specify the methods used to collect data from reports, including how many reviewers collected data from each report, whether they worked independently, any processes for obtaining or confirming data from study investigators, and if applicable, details of automation tools used in the process.	Search strategies paragraph
Data items	10a	List and define all outcomes for which data were sought. Specify whether all results that were compatible with each outcome domain in each study were sought (e.g. for all measures, time points, analyses), and if not, the methods used to decide which results to collect.	Screening of article paragraph

Section and Topic	Item #	Checklist item	Location where item is reported
	10b	List and define all other variables for which data were sought (e.g. participant and intervention characteristics, funding sources). Describe any assumptions made about any missing or unclear information.	Screening of article paragraph
Study risk of bias assessment	11	Specify the methods used to assess risk of bias in the included studies, including details of the tool(s) used, how many reviewers assessed each study and whether they worked independently, and if applicable, details of automation tools used in the process.	Quality assessment Paragraph
Effect measures	12	Specify for each outcome the effect measure(s) (e.g. risk ratio, mean difference) used in the synthesis or presentation of results.	Statistical analysis paragraph
Synthesis methods	13a	Describe the processes used to decide which studies were eligible for each synthesis (e.g. tabulating the study intervention characteristics and comparing against the planned groups for each synthesis (item #5)).	Table 1,2
	13b	Describe any methods required to prepare the data for presentation or synthesis, such as handling of missing summary statistics, or data conversions.	3rd paragraph
	13c	Describe any methods used to tabulate or visually display results of individual studies and syntheses.	Statistical analysis paragraph
	13d	Describe any methods used to synthesize results and provide a rationale for the choice(s). If meta-analysis was performed, describe the model(s), method(s) to identify the presence and extent of statistical heterogeneity, and software package(s) used.	Statistical analysis paragraph
	13e	Describe any methods used to explore possible causes of heterogeneity among study results (e.g. subgroup analysis, meta-regression).	Statistical analysis paragraph
	13f	Describe any sensitivity analyses conducted to assess robustness of the synthesized results.	Data synthesis last sentence
Reporting bias assessment	14	Describe any methods used to assess risk of bias due to missing results in a synthesis (arising from reporting biases).	Quality assessment Paragraph

Section and Topic	Item #	Checklist item	Location where item is reported
Certainty assessment	15	Describe any methods used to assess certainty (or confidence) in the body of evidence for an outcome.	Quality assessment Paragraph
**RESULTS**			
Study selection	16a	Describe the results of the search and selection process, from the number of records identified in the search to the number of studies included in the review, ideally using a flow diagram.	Study selection and screening paragraph
	16b	Cite studies that might appear to meet the inclusion criteria, but which were excluded, and explain why they were excluded.	Study selection and screening paragraph
Study characteristics	17	Cite each included study and present its characteristics.	Study characteristics paragraph
Risk of bias in studies	18	Present assessments of risk of bias for each included study.	figure 1
Results of individual studies	19	For all outcomes, present, for each study: (a) summary statistics for each group (where appropriate) and (b) an effect estimate and its precision (e.g. confidence/credible interval), ideally using structured tables or plots.	Result section
Results of syntheses	20a	For each synthesis, briefly summarise the characteristics and risk of bias among contributing studies.	Risk of bias and study quality paragraph
	20b	Present results of all statistical syntheses conducted. If meta-analysis was done, present for each the summary estimate and its precision (e.g. confidence/credible interval) and measures of statistical heterogeneity. If comparing groups, describe the direction of the effect.	Results paragraph and figure 1-4 and in the supplemental

Section and Topic	Item #	Checklist item	Location where item is reported
			file 4 - 12
	20c	Present results of all investigations of possible causes of heterogeneity among study results.	Publication bias and sensitivity analysis paragraph
	20d	Present results of all sensitivity analyses conducted to assess the robustness of the synthesized results.	Publication bias and sensitivity analysis paragraph
Reporting biases	21	Present assessments of risk of bias due to missing results (arising from reporting biases) for each synthesis assessed.	Publication bias and sensitivity analysis paragraph
Certainty of evidence	22	Present assessments of certainty (or confidence) in the body of evidence for each outcome assessed.	Publication bias and sensitivity analysis paragraph
**DISCUSSION**			
Discussion	23a	Provide a general interpretation of the results in the context of other evidence.	1st paragraph
	23b	Discuss any limitations of the evidence included in the review.	Limitation paragraph
	23c	Discuss any limitations of the review processes used.	Limitation and clinical implication
	23d	Discuss implications of the results for practice, policy, and future research.	Limitation

Section and Topic	Item #	Checklist item	Location where item is reported
			and clinical implication
**OTHER INFORMATION**			
Registration and protocol	24a	Provide registration information for the review, including register name and registration number, or state that the review was not registered.	Method 1st paragraph
	24b	Indicate where the review protocol can be accessed, or state that a protocol was not prepared.	N/A
	24c	Describe and explain any amendments to information provided at registration or in the protocol.	N/A
Support	25	Describe sources of financial or non-financial support for the review, and the role of the funders or sponsors in the review.	Fundings paragraph
Competing interests	26	Declare any competing interests of review authors.	Conflict of interest paragraph
Availability of data, code and other materials	27	Report which of the following are publicly available and where they can be found: template data collection forms; data extracted from included studies; data used for all analyses; analytic code; any other materials used in the review.	Supplemental file provided

From: Page MJ, McKenzie JE, Bossuyt PM, Boutron I, Hoffmann TC, Mulrow CD, et al. The PRISMA 2020 statement: an updated guideline for reporting systematic reviews. BMJ 2021;372:n71. doi: 10.1136/bmj.n71

Supplementary file 3: Risk of bias among included articles.

Study	Randomization process	Deviations from intended interventions	Missing outcome data	Measurement of the outcome	Selection of the reported result	Overall
Tang et al. 2013	+	+	+	+	+	+
Moore et al. 2014	+	+	+	+	−	−
Moore et al. 2016	+	+	+	+	+	+
Faulkner et al. 2016	+	+	+	+	−	−
Gambassi et al. 2019	×	+	+	+	×	×
Hus et al. 2020	+	−	+	+	−	−
Gjellesvik et al. 2020	+	+	+	+	×	−
Tollar et al. 2020	+	+	+	+	−	−
Kim et al. 2014	+	+	+	×	−	−
Lapointe et al. 2023	+	+	+	+	−	−
Deijle et al. 2022	+	+	−	+	−	−
Kang et al. 2023	+	+	+	+	+	+
Sakakibara et al. 2022	+	+	+	−	+	+
Krawcyk et al. 2019	+	+	+	+	+	+
Aguiar et al. 2020	+	+	+	+	+	+
Macko et al. 2005	+	−	+	−	+	−
Reynolds et al. 2021	+	+	+	−	−	−
Ribeiro et al. 2017	+	+	+	+	+	+

Sandberg et al. 2020	+	+	−	+	+	+
Wijkman et al. 2018	+	−	+	+	−	−
Acheampong et al. 2018	+	−	+	×	+	×
Globas et al. 2012	+	+	+	+	+	+
Han et al. 2017	+	+	+	+	−	+
Jin et al. 2013, China	−	×	+	+	−	×
Lee et al. 2013	−	+	+	+	−	−
Quaney et al. 2009	−	+	+	+	+	+
Sutbeyaze et al. 2008	+	+	+	+	+	+
Potempa et al. 1995	−	+	−	+	+	−
Zou et al. 2015	+	+	+	+	+	+
Stoller et al. 2015	+	+	+	+	+	+
Faulkner et al. 2013	+	+	+	+	+	+
Kirk et al. 2013	+	+	+	+	+	+
Kono et al. 2013	+	+	+	+	+	+
Lennon et al. 2008	+	+	+	+	+	+
MacKay-Lyons et al. 2022	+	+	+	+	+	+

+ Low Risk − Some Concerns × High Risk

Supplementary file 4: Subgroup analysis from baseline to post-intervention changes (A) systolic blood pressure, (B) diastolic blood pressure after rehabilitation, (C) Funnel Plot. (SD: Standard deviation; IV: Inverse-variance, CI: Confidence interval; df: degree of freedom)

A

Study or Subgroup	Baseline Mean	Baseline SD	Baseline Total	Post-interventation Mean	Post-interventation SD	Post-interventation Total	Weight	Mean Difference IV, Random, 95% CI
**1.3.1 SBP_Aerobic_Exercise**								
Deijle et al. 2022_A	124.5	15	60	124.2	17.3	60	7.9%	0.30 [-5.49, 6.09]
Fulkner et al. 2016_A	134	21	25	125	19	25	2.1%	9.00 [-2.10, 20.10]
Gjellesvik et al. 2020_A	138.46	16	35	135.7	13.62	33	5.3%	2.76 [-4.29, 9.81]
Jin et al. 2013_A	120.2	12.4	65	119.2	16.7	65	10.4%	1.00 [-4.06, 6.06]
Krawcyk et al. 2019_B	147	21	32	141	16	32	3.2%	6.00 [-3.15, 15.15]
Lapointe et al. 2023_A	130.3	17	16	128.9	12.8	16	2.4%	1.40 [-9.03, 11.83]
Lapointe et al. 2023_B	125.3	13.9	19	123.4	13.7	19	3.4%	1.90 [-6.88, 10.68]
Wijkman et al. 2017_A	140	23.4	29	135	18	29	2.3%	5.00 [-5.74, 15.74]
**Subtotal (95% CI)**			**281**			**279**	**37.0%**	**2.35 [-0.32, 5.03]**
Heterogeneity: Tau² = 0.00; Chi² = 3.03, df = 7 (P = 0.88); I² = 0%								
Test for overall effect: Z = 1.72 (P = 0.08)								
**1.3.2 SBP_ Resistance_Training**								
Deijle et al. 2022_B	123.2	15.2	59	122	13.2	59	10.0%	1.20 [-3.94, 6.34]
Gambassi et al. 2019_A	133	33.49	11	129	30.51	11	0.4%	4.00 [-22.77, 30.77]
Moore et al. 2014_A	137	27	20	143	19	20	1.3%	-6.00 [-20.47, 8.47]
**Subtotal (95% CI)**			**90**			**90**	**11.7%**	**0.51 [-4.26, 5.27]**
Heterogeneity: Tau² = 0.00; Chi² = 0.91, df = 2 (P = 0.63); I² = 0%								
Test for overall effect: Z = 0.21 (P = 0.83)								
**1.3.3 SBP_Standard_Care**								
Acheampong et al. 2018_B	136.5	8	8	142.13	8	8	4.3%	-5.63 [-13.47, 2.21]
Faulkner et al. 2013_B	137.9	12	30	134	15.5	30	5.4%	3.90 [-3.11, 10.91]
Fulkner et al. 2016_B	140	27	22	138	28	22	1.0%	2.00 [-14.25, 18.25]
Gambassi et al. 2019_B	133	33.49	11	129	30.51	11	0.4%	4.00 [-22.77, 30.77]
Gjellesvik et al. 2020_B	141	13.94	31	138.16	14.6	31	5.2%	2.84 [-4.27, 9.95]
Jin et al. 2013_B	119.7	13.2	63	118.9	16.9	63	9.4%	0.80 [-4.50, 6.10]
Kirk et al. 2013_B	134.33	16.3	12	131.92	18.1	12	1.4%	2.41 [-11.37, 16.19]
Kono et al. 2013_B	136.8	11.2	35	138.9	13.8	35	7.6%	-2.10 [-7.99, 3.79]
Lapointe et al. 2023_C	132.3	18.7	17	127.4	16	17	1.9%	4.90 [-6.80, 16.60]
Lennon et al. 2008_B	135.2	17.5	23	133.5	16.7	23	2.7%	1.70 [-8.19, 11.59]
MacKay-Lyons et al. 2022B	131.7	18.4	90	129.3	18.1	90	9.3%	2.40 [-2.93, 7.73]
Wijkman et al. 2017_B	139.6	20.4	24	133.8	15	24	2.6%	5.80 [-4.33, 15.93]
**Subtotal (95% CI)**			**366**			**366**	**51.3%**	**1.20 [-1.08, 3.47]**
Heterogeneity: Tau² = 0.00; Chi² = 6.38, df = 11 (P = 0.85); I² = 0%								
Test for overall effect: Z = 1.03 (P = 0.30)								
**Total (95% CI)**			**737**			**735**	**100.0%**	**1.54 [-0.08, 3.17]**
Heterogeneity: Tau² = 0.00; Chi² = 10.95, df = 22 (P = 0.98); I² = 0%								
Test for overall effect: Z = 1.86 (P = 0.06)								
Test for subgroup differences: Chi² = 0.62, df = 2 (P = 0.73), I² = 0%								

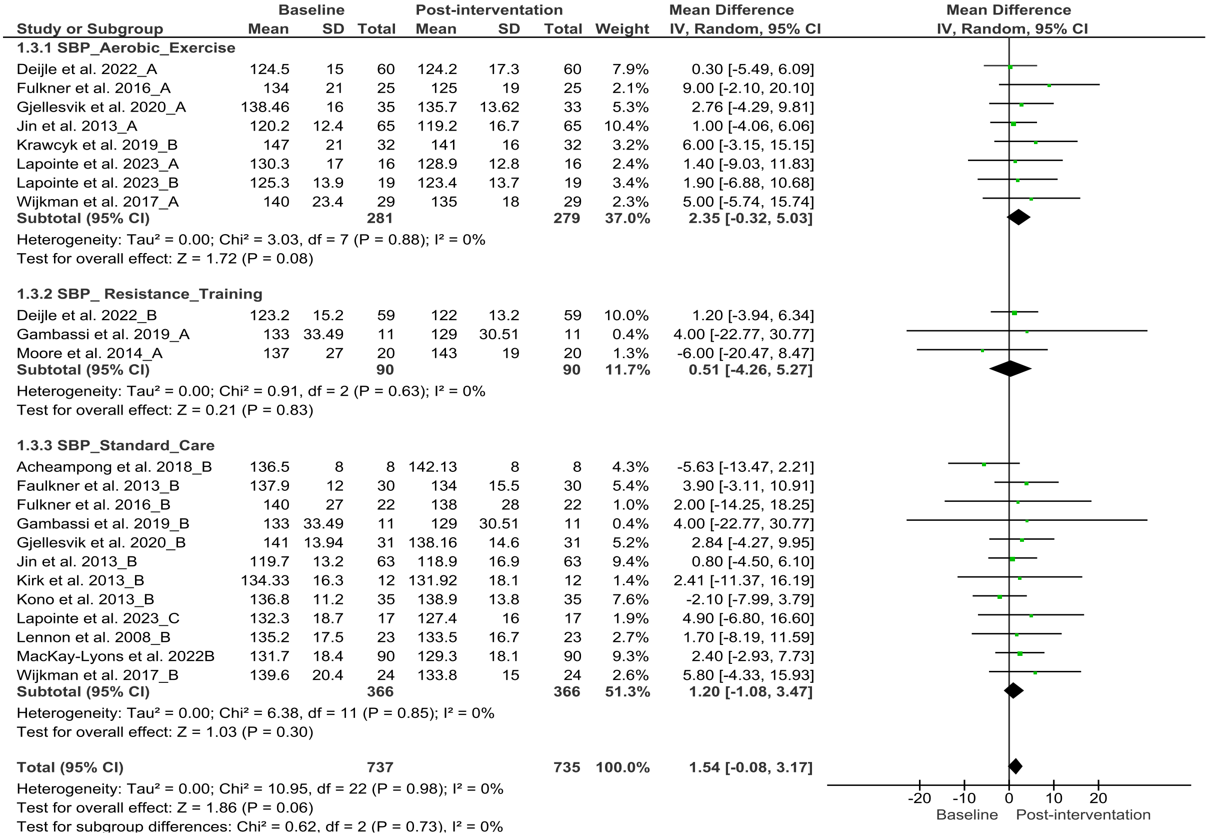

B

Study or Subgroup	Baseline Mean	Baseline SD	Baseline Total	Post-interventation Mean	Post-interventation SD	Post-interventation Total	Weight	Mean Difference IV, Random, 95% CI
**1.4.4 DBP_Aerobic_Exercise**								
Deijle et al. 2022_A	74.9	9.6	60	75.3	9.7	60	9.1%	-0.40 [-3.85, 3.05]
Fulkner et al. 2016_A	77	10	25	73	10	25	3.5%	4.00 [-1.54, 9.54]
Gjellesvik et al. 2020_A	83.14	11.58	35	83.42	7.35	33	5.2%	-0.28 [-4.86, 4.30]
Jin et al. 2013_A	75.8	11.2	65	75	10.2	65	8.0%	0.80 [-2.88, 4.48]
Krawcyk et al. 2019_B	83	10	32	83	10	32	4.5%	0.00 [-4.90, 4.90]
Lapointe et al. 2023_A	68	9.5	16	67.2	12	16	1.9%	0.80 [-6.70, 8.30]
Lapointe et al. 2023_B	65	12.3	19	65.3	7.8	19	2.5%	-0.30 [-6.85, 6.25]
Wijkman et al. 2017_A	77.1	7.7	29	74.8	7.8	29	6.8%	2.30 [-1.69, 6.29]
**Subtotal (95% CI)**			**281**			**279**	**41.6%**	**0.77 [-0.85, 2.38]**
Heterogeneity: Tau² = 0.00; Chi² = 2.71, df = 7 (P = 0.91); I² = 0%								
Test for overall effect: Z = 0.93 (P = 0.35)								
**1.4.5 DBP_Resistance_Training**								
Deijle et al. 2022_B	74.3	9.7	59	74.3	8	59	10.5%	0.00 [-3.21, 3.21]
Gambassi et al. 2019_A	79.2	11.9	11	79.8	10.9	11	1.2%	-0.60 [-10.14, 8.94]
Moore et al. 2014_A	84	10	20	81	11	20	2.6%	3.00 [-3.52, 9.52]
**Subtotal (95% CI)**			**90**			**90**	**14.3%**	**0.49 [-2.27, 3.24]**
Heterogeneity: Tau² = 0.00; Chi² = 0.71, df = 2 (P = 0.70); I² = 0%								
Test for overall effect: Z = 0.35 (P = 0.73)								
**1.4.6 DBP_Standard_Care**								
Acheampong et al. 2018_B	79.88	8	8	78.25	8	8	1.8%	1.63 [-6.21, 9.47]
Faulkner et al. 2013_B	80.4	8	30	79.5	16.13	30	2.6%	0.90 [-5.54, 7.34]
Fulkner et al. 2016_B	77	9	22	75	11	22	3.1%	2.00 [-3.94, 7.94]
Gambassi et al. 2019_B	79.2	11.9	11	79.8	10.9	11	1.2%	-0.60 [-10.14, 8.94]
Gjellesvik et al. 2020_B	87.15	8.34	34	86.59	9.73	31	5.5%	0.56 [-3.87, 4.99]
Jin et al. 2013_B	74	11.1	63	74.8	10.6	63	7.6%	-0.80 [-4.59, 2.99]
Kirk et al. 2013_B	76.67	7.9	12	74.67	8.7	12	2.5%	2.00 [-4.65, 8.65]
Kono et al. 2013_B	82.2	14.1	35	80.7	10.7	35	3.2%	1.50 [-4.36, 7.36]
Lapointe et al. 2023_C	70.7	9.6	17	73.3	10	17	2.5%	-2.60 [-9.19, 3.99]
Lennon et al. 2008_B	81.8	11.6	23	82	9	23	3.0%	-0.20 [-6.20, 5.80]
MacKay-Lyons et al. 2022B	74.9	9.8	90	73.7	14.6	90	8.2%	1.20 [-2.43, 4.83]
Wijkman et al. 2017_B	77.8	12.8	24	74.8	7.9	24	3.0%	3.00 [-3.02, 9.02]
**Subtotal (95% CI)**			**369**			**366**	**44.1%**	**0.66 [-0.91, 2.23]**
Heterogeneity: Tau² = 0.00; Chi² = 2.82, df = 11 (P = 0.99); I² = 0%								
Test for overall effect: Z = 0.83 (P = 0.41)								
**Total (95% CI)**			**740**			**735**	**100.0%**	**0.68 [-0.36, 1.72]**
Heterogeneity: Tau² = 0.00; Chi² = 6.27, df = 22 (P = 1.00); I² = 0%								
Test for overall effect: Z = 1.28 (P = 0.20)								
Test for subgroup differences: Chi² = 0.03, df = 2 (P = 0.98), I² = 0%								

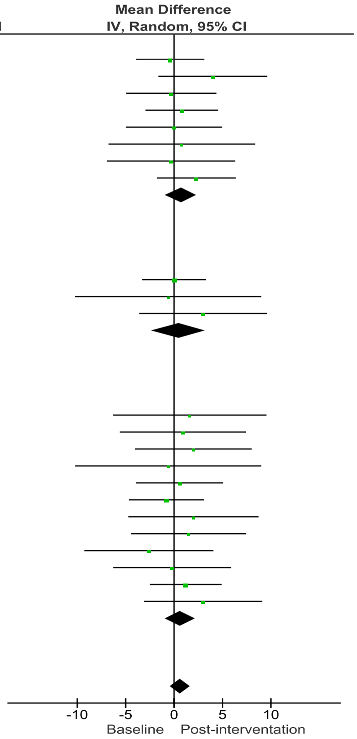

C

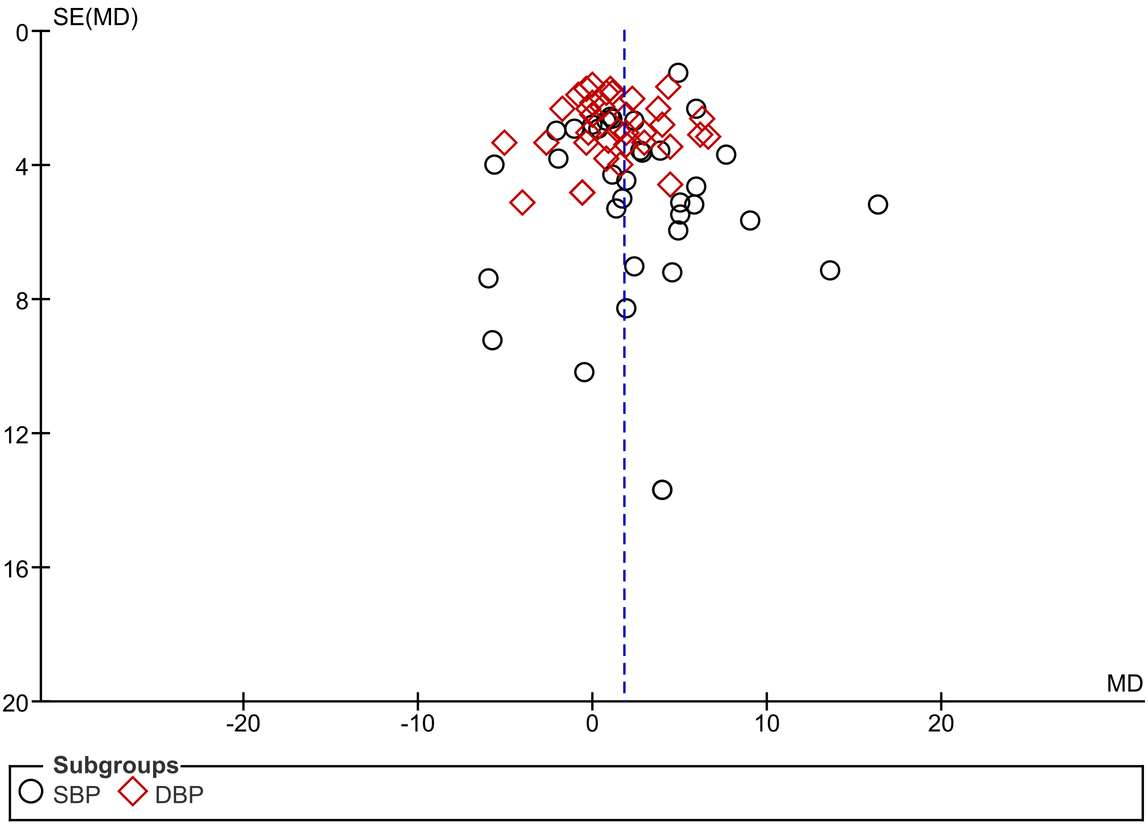

Supplementary file 5: Cardiac output changes (A) baseline to post-intervention, (B) difference in pre-and post-intervention at control and intervention groups after rehabilitation. (SD: Standard deviation; IV: Inverse-variance, CI: Confidence interval; df: degree of freedom)

A

Study or Subgroup	Baseline Mean	Baseline SD	Baseline Total	Post-intervention Mean	Post-intervention SD	Post-intervention Total	Weight	Mean Difference IV, Random, 95% CI
Hus et al. 2020_A	8.35	1.98	10	9.8	2.94	10	10.2%	-1.45 [-3.65, 0.75]
Hus et al. 2020_B	9.78	1.68	13	10.1	2.98	13	14.3%	-0.32 [-2.18, 1.54]
Moore et al. 2016_A	5.6	1.2	20	5.54	1.4	20	75.5%	0.06 [-0.75, 0.87]
**Total (95% CI)**			**43**			**43**	**100.0%**	**-0.15 [-0.85, 0.55]**

Heterogeneity: Tau² = 0.00; Chi² = 1.64, df = 2 (P = 0.44); I² = 0%
Test for overall effect: Z = 0.41 (P = 0.68)

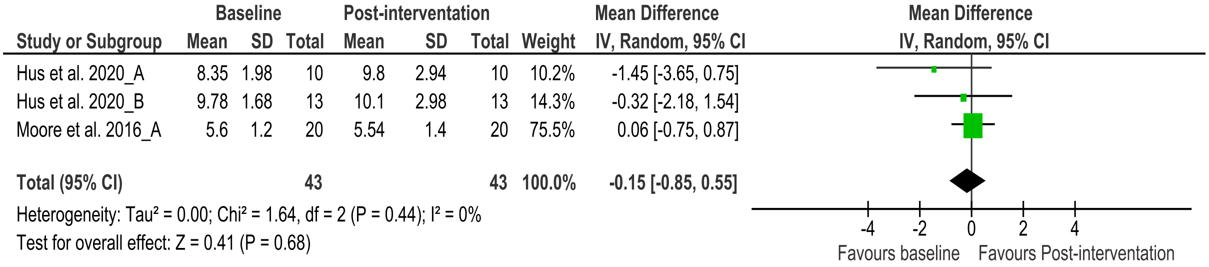

B

Study or Subgroup	Control Mean	Control SD	Control Total	Intervention Mean	Intervention SD	Intervention Total	Weight	Mean Difference IV, Random, 95% CI
Hus et al. 2020	0.32	2.6	13	1.45	2.6	10	8.9%	-1.13 [-3.27, 1.01]
Moore et al. 2014	-0.38	0.8	20	-0.06	1.3	20	91.1%	-0.32 [-0.99, 0.35]
**Total (95% CI)**			**33**			**30**	**100.0%**	**-0.39 [-1.03, 0.25]**

Heterogeneity: Tau² = 0.00; Chi² = 0.50, df = 1 (P = 0.48); I² = 0%
Test for overall effect: Z = 1.20 (P = 0.23)

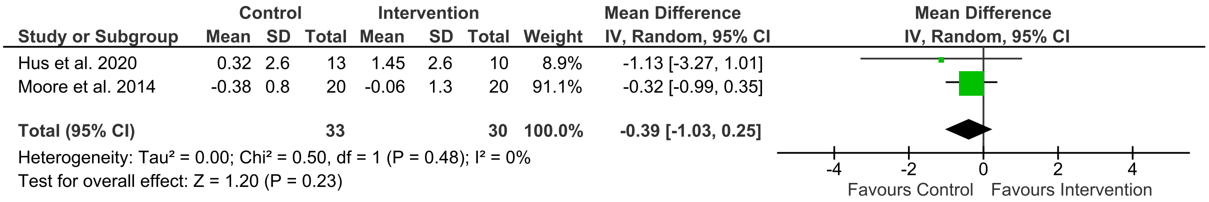

Supplementary file 6: (A) High-density lipoprotein changes from baseline to post-intervention, (B) difference in control and intervention groups, (C) HDL Funnel Plot, (D) LDL Funnel Plot. (SD: Standard deviation; IV: Inverse-variance, CI: Confidence interval; df: degree of freedom)

A

	Baseline			Post-interventation				Mean Difference
**Study or Subgroup**	**Mean**	**SD**	**Total**	**Mean**	**SD**	**Total**	**Weight**	**IV, Random, 95% CI**
Acheampong et al. 2018_A	1.23	0.26	5	1.17	0.23	5	1.0%	0.06 [-0.24, 0.36]
Acheampong et al. 2018_B	1.22	8	8	1.21	8	8	0.0%	0.01 [-7.83, 7.85]
Faulkner et al. 2013_A	1.31	0.6	30	1.38	0.63	30	0.9%	-0.07 [-0.38, 0.24]
Faulkner et al. 2013_B	1.28	0.52	30	1.43	0.55	30	1.2%	-0.15 [-0.42, 0.12]
Fulkner et al. 2016_A	1.11	0.3	25	1.15	0.32	25	3.1%	-0.04 [-0.21, 0.13]
Fulkner et al. 2016_B	1.13	0.3	22	1.15	0.32	22	2.7%	-0.02 [-0.20, 0.16]
Gjellesvik et al. 2020_A	1.43	0.44	33	1.44	0.44	33	2.0%	-0.01 [-0.22, 0.20]
Gjellesvik et al. 2020_B	1.49	0.48	34	1.51	0.47	29	1.6%	-0.02 [-0.26, 0.22]
Kirk et al. 2013_A	1.25	0.3	12	1.22	0.3	12	1.6%	0.03 [-0.21, 0.27]
Kirk et al. 2013_B	1.1	0.3	12	1.1	0.2	12	2.2%	0.00 [-0.20, 0.20]
Krawcyk et al. 2019_A	1.4	0.5	31	1.5	0.5	31	1.5%	-0.10 [-0.35, 0.15]
Krawcyk et al. 2019_B	1.4	0.4	32	1.4	0.4	32	2.4%	0.00 [-0.20, 0.20]
Lapointe et al. 2023_A	1.3	0.4	16	1.4	0.4	16	1.2%	-0.10 [-0.38, 0.18]
Lapointe et al. 2023_B	1.4	0.6	19	1.4	0.5	19	0.7%	0.00 [-0.35, 0.35]
Lapointe et al. 2023_C	1.3	0.3	17	1.3	0.3	17	2.2%	0.00 [-0.20, 0.20]
MacKay-Lyons et al. 2022A	1.16	0.4	94	1.24	0.4	94	6.9%	-0.08 [-0.19, 0.03]
MacKay-Lyons et al. 2022B	1.15	0.3	90	1.17	0.3	90	11.8%	-0.02 [-0.11, 0.07]
Moore et al. 2014_A	1.3	0.4	20	1.6	0.6	20	0.9%	-0.30 [-0.62, 0.02]
Moore et al. 2014_B	1.4	0.4	20	1.4	0.3	20	1.9%	0.00 [-0.22, 0.22]
Sakakibara et al. 2022_A	1.5	0.4	64	1.5	0.4	64	4.7%	0.00 [-0.14, 0.14]
Sakakibara et al. 2022_B	1.4	0.4	61	1.4	0.4	62	4.5%	0.00 [-0.14, 0.14]
Tang et al. 2013_A	1.4	0.4	22	1.4	0.3	22	2.1%	0.00 [-0.21, 0.21]
Tang et al. 2013_B	1.3	0.3	25	1.3	0.4	25	2.4%	0.00 [-0.20, 0.20]
Zou et al. 2015_B	1.14	0.09	28	1.15	0.09	28	40.7%	-0.01 [-0.06, 0.04]
**Total (95% CI)**			**750**			**746**	**100.0%**	**-0.02 [-0.05, 0.01]**

Heterogeneity: Tau² = 0.00; Chi² = 6.83, df = 23 (P = 1.00); I² = 0%
Test for overall effect: Z = 1.37 (P = 0.17)

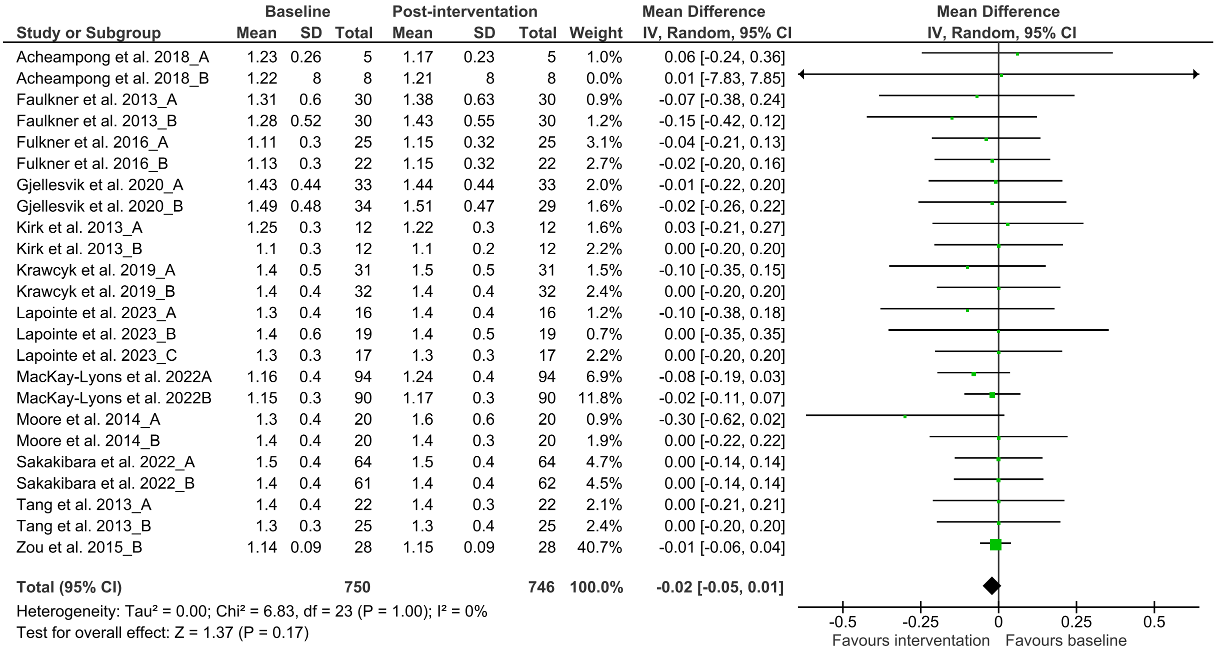

B

	Control			Intervention				Mean Difference
**Study or Subgroup**	**Mean**	**SD**	**Total**	**Mean**	**SD**	**Total**	**Weight**	**IV, Random, 95% CI**
Acheampong et al. 2018	-0.01	8	8	-0.06	0.2	5	0.0%	0.05 [-5.50, 5.60]
Faulkner et al. 2013	0.15	0.53	30	0.07	0.61	30	3.8%	0.08 [-0.21, 0.37]
Fulkner et al. 2016	0.02	0.3	22	0.04	0.3	25	10.7%	-0.02 [-0.19, 0.15]
Gjellesvik et al. 2020	0.02	0.5	29	0.01	0.4	33	6.1%	0.01 [-0.22, 0.24]
Kirk et al. 2013	0	0.26	12	-0.03	0.3	12	6.3%	0.03 [-0.19, 0.25]
Krawcyk et al. 2019	0	0.4	32	0.1	0.5	31	6.3%	-0.10 [-0.32, 0.12]
Lapointe et al. 2023	0	0.3	17	0.1	0.4	16	5.4%	-0.10 [-0.34, 0.14]
Lapointe et al. 2023_2	0	0.3	17	0	0.5	19	4.5%	0.00 [-0.27, 0.27]
MacKay-Lyons et al. 2022	0.02	0.3	90	0.08	0.4	94	30.5%	-0.06 [-0.16, 0.04]
Moore et al. 2014	0	0.4	20	0.3	0.5	20	4.0%	-0.30 [-0.58, -0.02]
Sakakibara et al. 2022	0	0.4	62	0	0.4	64	16.2%	0.00 [-0.14, 0.14]
Tang et al. 2013	0	0.4	25	0	0.4	22	6.0%	0.00 [-0.23, 0.23]
**Total (95% CI)**			**364**			**371**	**100.0%**	**-0.04 [-0.10, 0.02]**

Heterogeneity: Tau² = 0.00; Chi² = 5.75, df = 11 (P = 0.89); I² = 0%
Test for overall effect: Z = 1.35 (P = 0.18)

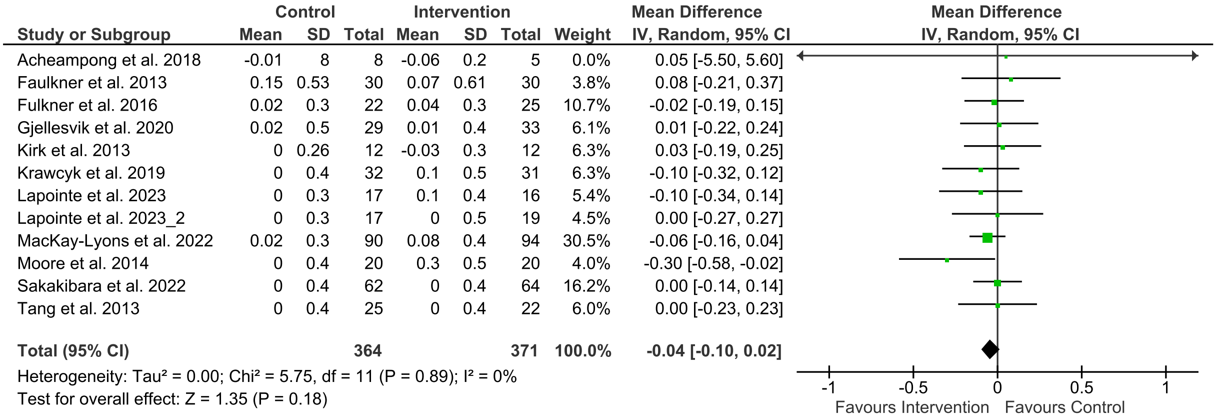

C

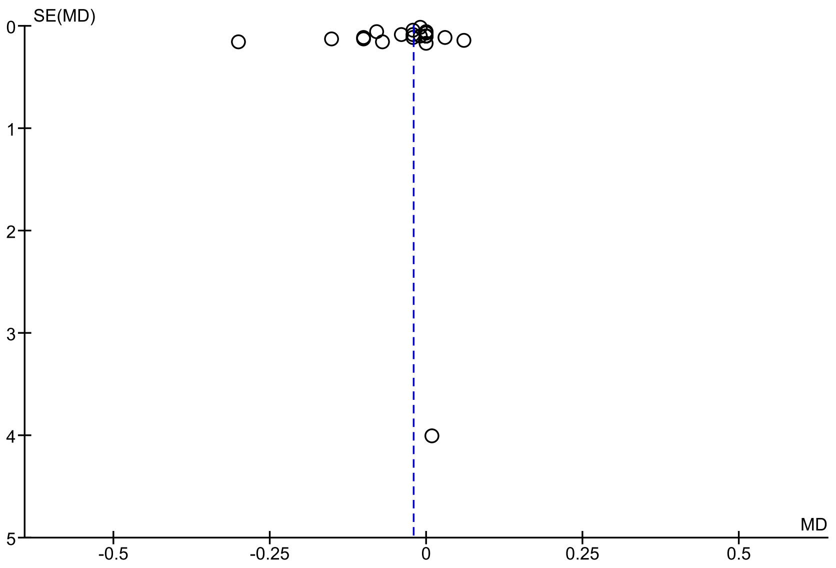

D

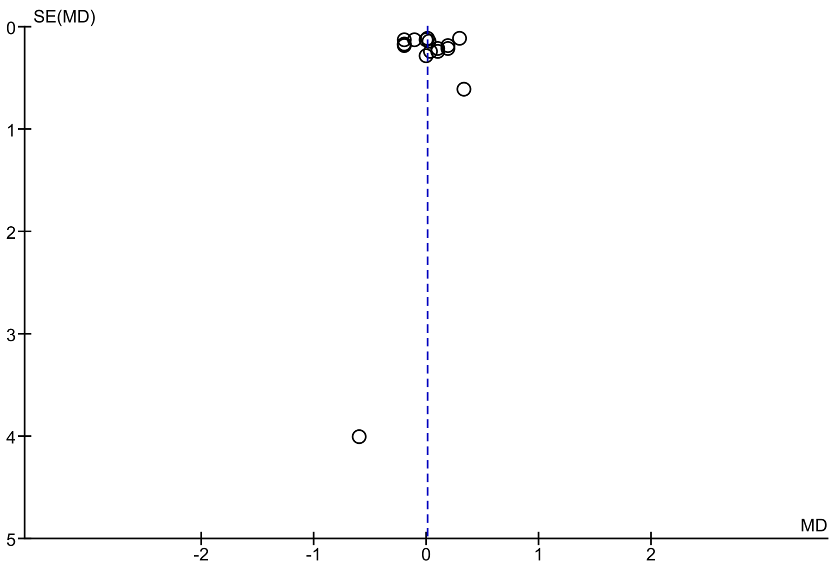

Supplementary file 7: Total cholesterol changes (A) baseline to post-intervention, (B) difference in pre-and post-intervention at control and intervention groups after rehabilitation, (C) Funnel plot. (SD: Standard deviation; IV: Inverse-variance, CI: Confidence interval; df: degree of freedom)

A

Study or Subgroup	Baseline Mean	Baseline SD	Baseline Total	Post-interventation Mean	Post-interventation SD	Post-interventation Total	Weight	Mean Difference IV, Random, 95% CI
Acheampong et al. 2018_A	3.77	1.08	5	3.46	0.87	5	0.9%	0.31 [-0.91, 1.53]
Acheampong et al. 2018_B	5.15	8	8	5.75	8	8	0.0%	-0.60 [-8.44, 7.24]
Deijle et al. 2022_A	4.4	0.8	60	4.5	1	60	12.9%	-0.10 [-0.42, 0.22]
Deijle et al. 2022_B	4.4	0.9	59	4.4	1	59	11.5%	0.00 [-0.34, 0.34]
Faulkner et al. 2013_A	4.14	1.22	30	3.7	0.9	30	4.6%	0.44 [-0.10, 0.98]
Faulkner et al. 2013_B	4.1	1.09	30	4.06	0.96	30	5.0%	0.04 [-0.48, 0.56]
Fulkner et al. 2016_A	4.04	1.07	25	3.81	1.05	25	3.9%	0.23 [-0.36, 0.82]
Fulkner et al. 2016_B	4.17	0.97	22	3.97	1	22	4.0%	0.20 [-0.38, 0.78]
Gjellesvik et al. 2020_A	4.3	1.06	33	4.12	0.96	33	5.7%	0.18 [-0.31, 0.67]
Gjellesvik et al. 2020_B	4.52	1.28	34	4.58	0.94	29	4.5%	-0.06 [-0.61, 0.49]
Kirk et al. 2013_A	3.95	3.7	12	3.8	3.6	12	0.2%	0.15 [-2.77, 3.07]
Kirk et al. 2013_B	3.75	3.5	12	3.55	3.7	12	0.2%	0.20 [-2.68, 3.08]
Lennon et al. 2008_A	4.6	1.1	23	4.5	1	23	3.7%	0.10 [-0.51, 0.71]
Lennon et al. 2008_B	4.6	1.2	23	4.7	1.1	23	3.1%	-0.10 [-0.77, 0.57]
Moore et al. 2014_A	4.5	1.2	20	4.7	1.2	20	2.5%	-0.20 [-0.94, 0.54]
Moore et al. 2014_B	4.4	0.8	20	4.2	0.8	20	5.5%	0.20 [-0.30, 0.70]
Tang et al. 2013_A	4.5	0.9	22	4.4	0.9	22	4.8%	0.10 [-0.43, 0.63]
Tang et al. 2013_B	4.3	0.9	25	4	0.9	25	5.5%	0.30 [-0.20, 0.80]
Zou et al. 2015_B	5.33	0.51	28	5.41	0.45	28	21.4%	-0.08 [-0.33, 0.17]
**Total (95% CI)**			**491**			**486**	**100.0%**	**0.05 [-0.07, 0.16]**

Heterogeneity: Tau² = 0.00; Chi² = 7.18, df = 18 (P = 0.99); I² = 0%
Test for overall effect: Z = 0.81 (P = 0.42)

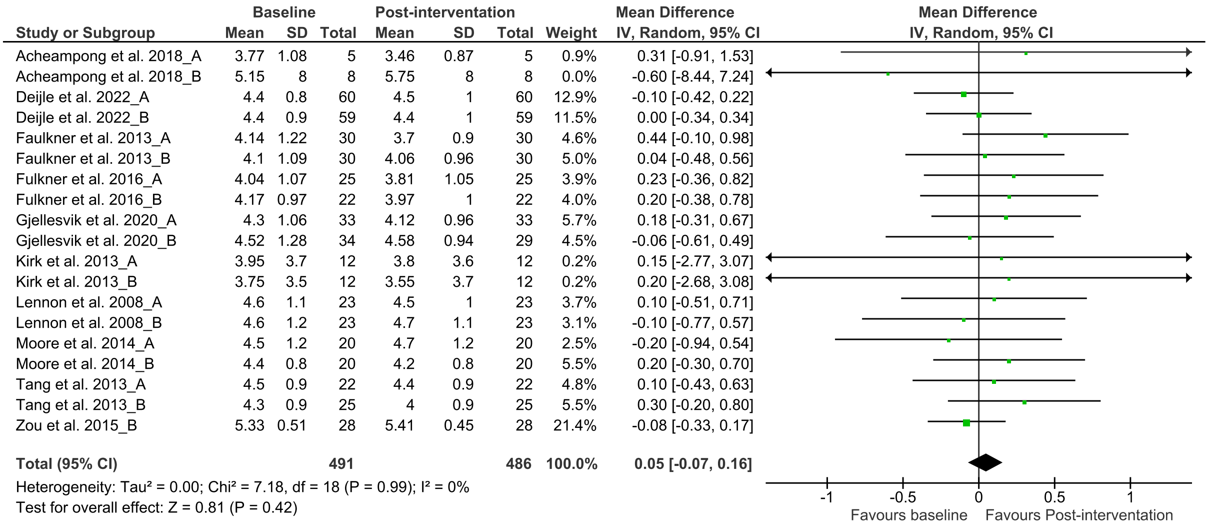

B

Study or Subgroup	Control Mean	Control SD	Control Total	Intervention Mean	Intervention SD	Intervention Total	Weight	Mean Difference IV, Random, 95% CI
Acheampong et al. 2018	0.6	8	8	-0.31	0.99	5	0.1%	0.91 [-4.70, 6.52]
Deijle et al. 2022	0	0.9	59	0.1	0.9	60	30.6%	-0.10 [-0.42, 0.22]
Faulkner et al. 2013	-0.04	1.03	30	-0.44	1.09	30	11.1%	0.40 [-0.14, 0.94]
Fulkner et al. 2016	-0.2	0.9	22	-0.23	1.1	25	9.8%	0.03 [-0.54, 0.60]
Gjellesvik et al. 2020	0.06	1.1	29	-0.18	1	33	11.6%	0.24 [-0.29, 0.77]
Kirk et al. 2013	-0.2	3.6	12	-0.15	3.65	12	0.4%	-0.05 [-2.95, 2.85]
Krawcyk et al. 2019	-1.4	1.31	32	-1.4	1.15	31	8.6%	0.00 [-0.61, 0.61]
Lennon et al. 2008	0.1	1.15	23	-0.1	1.05	23	7.9%	0.20 [-0.44, 0.84]
Moore et al. 2014	-0.2	0.8	20	0.2	1.2	20	8.0%	-0.40 [-1.03, 0.23]
Tang et al. 2013	-0.3	0.9	25	-0.1	0.9	22	12.0%	-0.20 [-0.72, 0.32]
**Total (95% CI)**			**260**			**261**	**100.0%**	**0.00 [-0.17, 0.18]**

Heterogeneity: Tau² = 0.00; Chi² = 5.91, df = 9 (P = 0.75); I² = 0%
Test for overall effect: Z = 0.05 (P = 0.96)

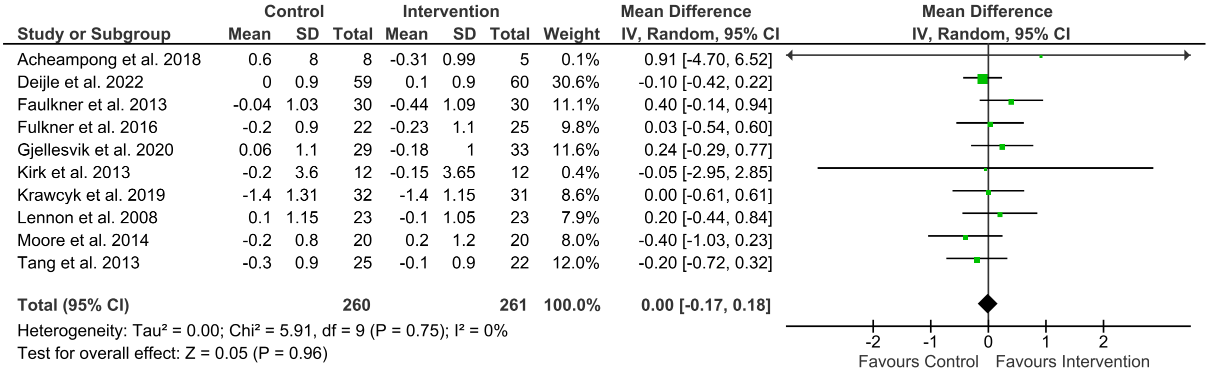

C

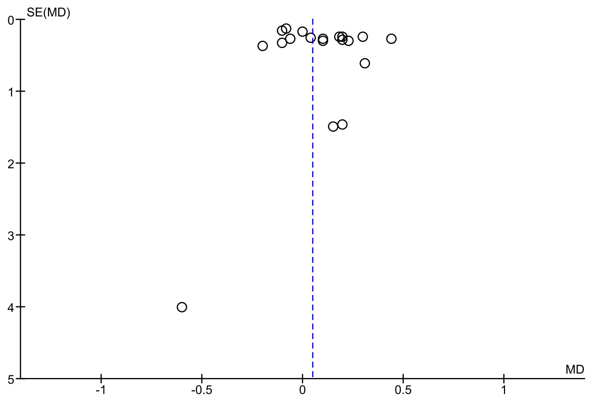

Supplementary file 8: Triglycerides changes (A) baseline to post-intervention, (B) difference in pre- and post-intervention at control and intervention groups after rehabilitation, (C) Funnel Plot. (SD: Standard deviation; IV: Inverse-variance, CI: Confidence interval; df: degree of freedom)

A

Study or Subgroup	Baseline Mean	Baseline SD	Baseline Total	Post-interventation Mean	Post-interventation SD	Post-interventation Total	Weight	Mean Difference IV, Random, 95% CI
Acheampong et al. 2018_A	1.11	0.28	5	1.05	0.23	5	7.0%	0.06 [-0.26, 0.38]
Acheampong et al. 2018_B	1.61	8	8	1.73	8	8	0.0%	-0.12 [-7.96, 7.72]
Gjellesvik et al. 2020_A	1.32	0.63	33	1.24	0.53	33	8.9%	0.08 [-0.20, 0.36]
Gjellesvik et al. 2020_B	1.45	1.45	34	1.51	1.63	29	1.2%	-0.06 [-0.83, 0.71]
Krawcyk et al. 2019_B	1.7	0.8	32	1.2	0.7	32	5.2%	0.50 [0.13, 0.87]
MacKay-Lyons et al. 2022A	1.4	0.8	94	1.22	0.7	94	15.2%	0.18 [-0.03, 0.39]
MacKay-Lyons et al. 2022B	1.41	0.8	90	1.38	0.7	90	14.6%	0.03 [-0.19, 0.25]
Tang et al. 2013_A	1.4	0.4	22	1.4	0.4	22	12.6%	0.00 [-0.24, 0.24]
Tang et al. 2013_B	1.6	0.8	25	1.3	0.5	25	5.1%	0.30 [-0.07, 0.67]
Zou et al. 2015_A	1.45	0.42	28	1.4	0.44	28	13.8%	0.05 [-0.18, 0.28]
Zou et al. 2015_B	1.46	0.39	28	1.41	0.4	28	16.4%	0.05 [-0.16, 0.26]
**Total (95% CI)**			**399**			**394**	**100.0%**	**0.10 [0.01, 0.18]**

Heterogeneity: Tau² = 0.00; Chi² = 7.93, df = 10 (P = 0.64); I² = 0%
Test for overall effect: Z = 2.31 (P = 0.02)

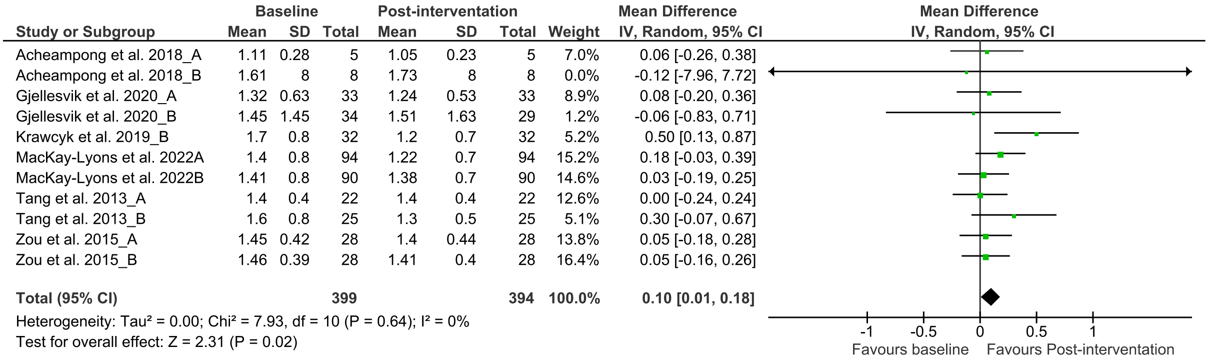

B

Study or Subgroup	Control Mean	Control SD	Control Total	Intervention Mean	Intervention SD	Intervention Total	Weight	Mean Difference IV, Random, 95% CI
Acheampong et al. 2018	0.12	8	8	-0.06	0.2	5	0.1%	0.18 [-5.37, 5.73]
Gjellesvik et al. 2020	0.06	1.5	29	-0.1	0.6	33	5.5%	0.16 [-0.42, 0.74]
Krawcyk et al. 2019	-0.5	0.75	32	-0.8	0.95	31	10.5%	0.30 [-0.12, 0.72]
MacKay-Lyons et al. 2022	-0.03	0.75	90	-0.18	0.75	94	40.0%	0.15 [-0.07, 0.37]
Zou et al. 2015	-0.05	0.39	28	-0.05	0.4	28	43.9%	0.00 [-0.21, 0.21]
**Total (95% CI)**			**187**			**191**	**100.0%**	**0.10 [-0.04, 0.24]**

Heterogeneity: Tau² = 0.00; Chi² = 2.00, df = 4 (P = 0.74); I² = 0%
Test for overall effect: Z = 1.44 (P = 0.15)

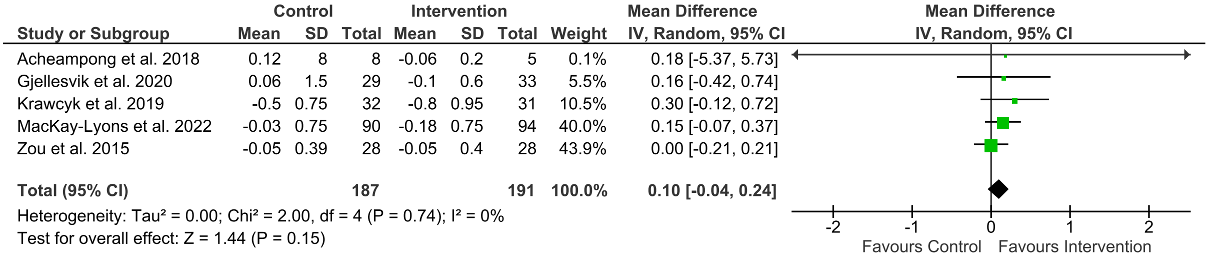

C

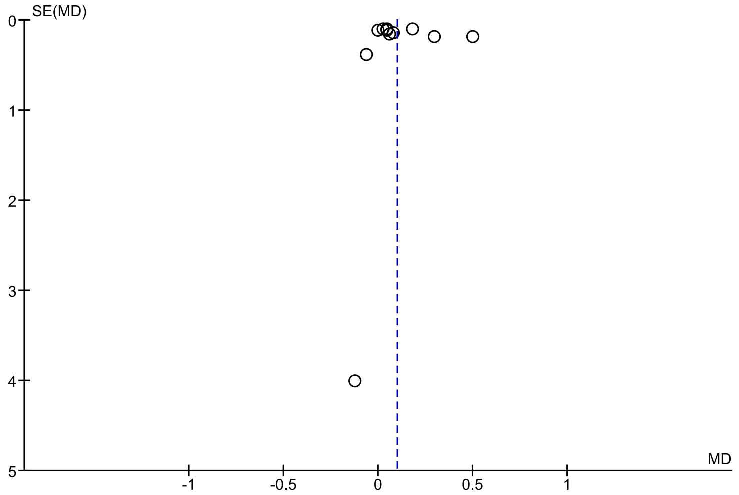

Supplementary file 9: 6MWT changes (A) baseline to post-intervention, and (B) difference in pre- and post-intervention at control and intervention groups after poststroke rehabilitation programs, (C) Funnel Plot. (SD: Standard deviation; IV: Inverse-variance, CI: Confidence interval; df: degree of freedom)

A

	Baseline			Post-interventation				Mean Difference
**Study or Subgroup**	**Mean**	**SD**	**Total**	**Mean**	**SD**	**Total**	**Weight**	**IV, Random, 95% CI**
Aguiar et al. 2020_A	378	79	11	322	120	11	3.4%	56.00 [-28.90, 140.90]
Aguiar et al. 2020_B	427	94	11	460	111	11	3.4%	-33.00 [-118.96, 52.96]
Globas et al. 2012_A	274.4	113	18	332.1	138	18	3.6%	-57.70 [-140.10, 24.70]
Globas et al. 2012_B	261.2	177	18	265.9	189	18	2.0%	-4.70 [-124.32, 114.92]
Han et al. 2017_A	289.2	90.05	10	328.6	64.62	10	4.7%	-39.40 [-108.10, 29.30]
Han et al. 2017_B	237.1	107.22	10	325.7	99.63	10	3.1%	-88.60 [-179.32, 2.12]
Jin et al. 2013_A	212.5	64.2	65	219.4	64.3	65	12.1%	-6.90 [-28.99, 15.19]
Jin et al. 2013_B	212.4	51.1	63	213.7	51.7	63	12.9%	-1.30 [-19.25, 16.65]
Kang et al. 2023_A	176.34	125.47	6	229.05	136.02	6	1.3%	-52.71 [-200.78, 95.36]
Kang et al. 2023_B	258.66	72.9	10	252.24	110.49	10	3.6%	6.42 [-75.62, 88.46]
Lee et al. 2013_A	193.63	102.97	8	266.25	113.38	8	2.4%	-72.62 [-178.75, 33.51]
Lee et al. 2013_B	291	82.61	8	331.5	60.22	8	4.5%	-40.50 [-111.34, 30.34]
Macko et al. 2005_B	848	109	29	868	100	20	5.7%	-20.00 [-79.11, 39.11]
Moore et al. 2014_B	419	127	20	441	126	20	3.9%	-22.00 [-100.40, 56.40]
Reynolds et al. 2021_A	400.9	111.6	10	443.3	126.4	10	2.5%	-42.40 [-146.91, 62.11]
Reynolds et al. 2021_B	344.8	150.6	10	370.9	126	10	1.9%	-26.10 [-147.80, 95.60]
Tang et al. 2013_A	278.2	128.5	10	298.1	134.2	22	2.8%	-19.90 [-117.31, 77.51]
Tang et al. 2013_B	322.2	142.4	25	331.5	149.2	25	3.7%	-9.30 [-90.15, 71.55]
Tollar et al. 2020_C	177.4	50.65	83	242.1	59.26	83	13.2%	-64.70 [-81.47, -47.93]
Yim et al. 2014_A	163.6	63.87	10	219.1	91.63	10	4.6%	-55.50 [-124.73, 13.73]
Yim et al. 2014_B	177.5	78.39	10	191.2	77.04	10	4.7%	-13.70 [-81.82, 54.42]
**Total (95% CI)**			**445**			**448**	**100.0%**	**-27.15 [-45.11, -9.18]**

Heterogeneity: Tau² = 565.39; Chi² = 39.38, df = 20 (P = 0.006); I² = 49%
Test for overall effect: Z = 2.96 (P = 0.003)

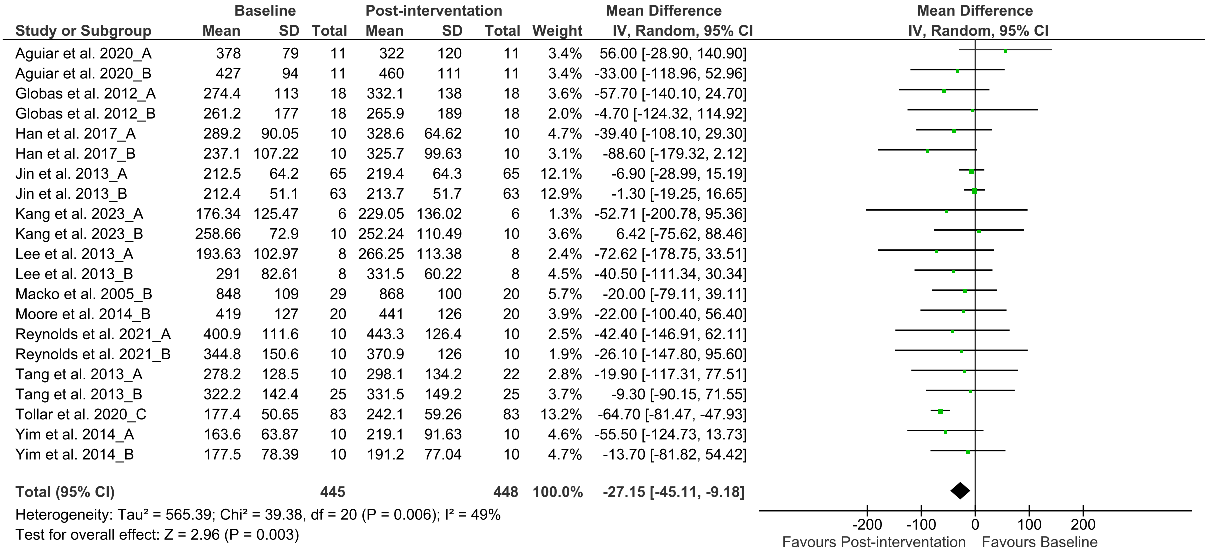

B

	Control			Intervention				Mean Difference
**Study or Subgroup**	**Mean**	**SD**	**Total**	**Mean**	**SD**	**Total**	**Weight**	**IV, Random, 95% CI**
Aguiar et al. 2020	33	103.55	11	-56	105.64	11	7.3%	89.00 [1.58, 176.42]
Globas et al. 2012	4.7	183.29	18	57.7	127.35	18	5.5%	-53.00 [-156.11, 50.11]
Han et al. 2017	88.6	103.63	10	39.4	80.41	10	8.2%	49.20 [-32.10, 130.50]
Jin et al. 2013	1.3	51.4	63	6.9	64.25	65	30.5%	-5.60 [-25.73, 14.53]
Kang et al. 2023	-6.42	97.3	10	52.7	131.1	6	4.2%	-59.12 [-180.12, 61.88]
Lee et al. 2013	40.5	74	8	72.62	108.55	8	6.8%	-32.12 [-123.16, 58.92]
Moore et al. 2014	22	126.5	20	85	2.6	20	14.0%	-63.00 [-118.45, -7.55]
Reynolds et al. 2021	26.1	139.93	10	42.4	119.69	10	4.7%	-16.30 [-130.43, 97.83]
Tang et al. 2013	9.3	145.9	25	19.9	131.4	22	8.5%	-10.60 [-89.88, 68.68]
Yim et al. 2014	13.7	77.7	10	55.5	81.4	10	10.3%	-41.80 [-111.55, 27.95]
**Total (95% CI)**			**185**			**180**	**100.0%**	**-13.61 [-39.95, 12.73]**

Heterogeneity: Tau² = 487.50; Chi² = 12.99, df = 9 (P = 0.16); I² = 31%
Test for overall effect: Z = 1.01 (P = 0.31)

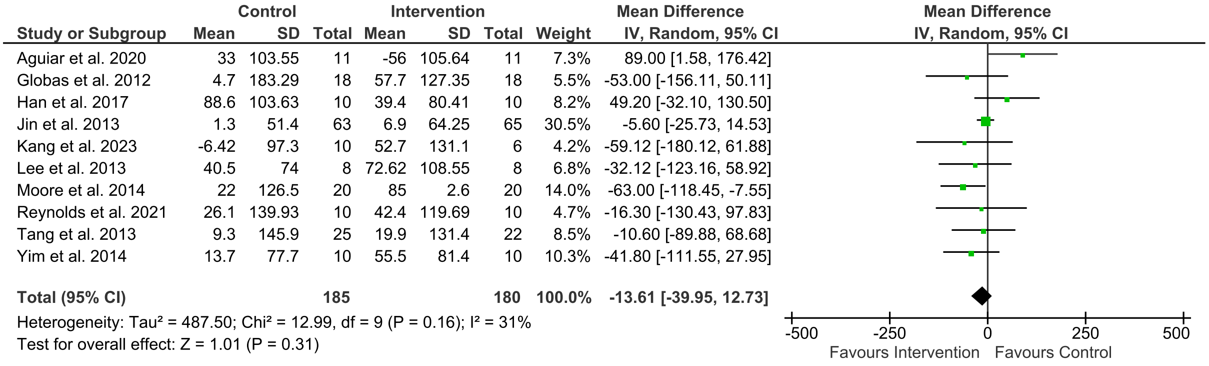

C

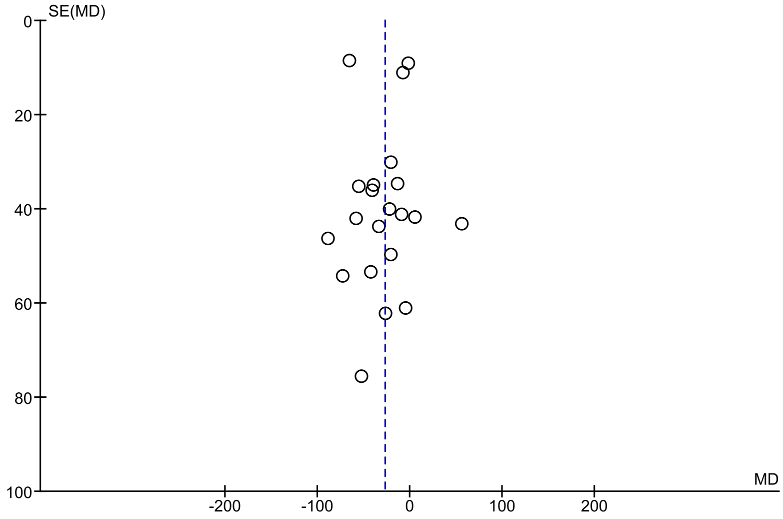

Supplementary file 10: Berg Balance score changes (A) baseline to post-intervention, and (B) difference in pre-and post-intervention at control and intervention groups after poststroke rehabilitation programs. (SD: Standard deviation; IV: Inverse-variance, CI: Confidence interval; df: degree of freedom)

A

	Baseline			Post-interventation				Mean Difference
**Study or Subgroup**	**Mean**	**SD**	**Total**	**Mean**	**SD**	**Total**	**Weight**	**IV, Random, 95% CI**
Moore et al. 2014_A	50	4	20	55	2	20	33.7%	-5.00 [-6.96, -3.04]
Moore et al. 2014_B	50	5.5	20	52	5	20	18.3%	-2.00 [-5.26, 1.26]
Tollar et al. 2020_C	22.1	4.42	83	24.9	3.41	83	48.0%	-2.80 [-4.00, -1.60]
**Total (95% CI)**			**123**			**123**	**100.0%**	**-3.39 [-5.04, -1.75]**

Heterogeneity: Tau² = 1.09; Chi² = 4.16, df = 2 (P = 0.12); I² = 52%
Test for overall effect: Z = 4.04 (P < 0.0001)

Mean Difference
IV, Random, 95% CI

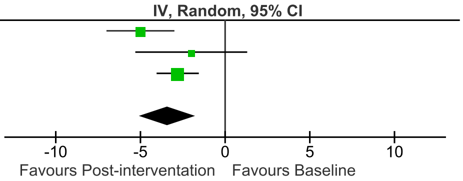

B

	Control			Intervention				Mean Difference
**Study or Subgroup**	**Mean**	**SD**	**Total**	**Mean**	**SD**	**Total**	**Weight**	**IV, Random, 95% CI**
Moore et al. 2014	2	5.3	20	5	3.5	20	100.0%	-3.00 [-5.78, -0.22]
**Total (95% CI)**			**20**			**20**	**100.0%**	**-3.00 [-5.78, -0.22]**

Heterogeneity: Not applicable
Test for overall effect: Z = 2.11 (P = 0.03)

Mean Difference
IV, Random, 95% CI

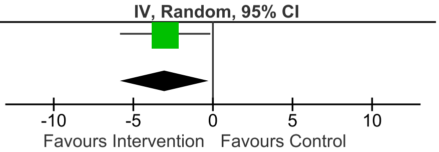

Supplementary file 11: Time-up and go score changes (A) baseline to post-intervention, and (B) difference in pre-and post-intervention at control and intervention groups after poststroke rehabilitation programs. (SD: Standard deviation; IV: Inverse-variance, CI: Confidence interval; df: degree of freedom)

A

	Baseline			Post-interventation				Mean Difference
Study or Subgroup	Mean	SD	Total	Mean	SD	Total	Weight	IV, Random, 95% CI
Gambassi et al. 2019_A	19.2	8.3	11	14.1	5.6	11	14.4%	5.10 [-0.82, 11.02]
Gambassi et al. 2019_B	22.2	9.3	11	22	7.1	11	10.6%	0.20 [-6.71, 7.11]
Moore et al. 2016_A	11	9	20	8.4	6	20	22.5%	2.60 [-2.14, 7.34]
Moore et al. 2016_B	9.8	5	20	9	5	20	52.6%	0.80 [-2.30, 3.90]
**Total (95% CI)**			**62**			**62**	**100.0%**	**1.76 [-0.49, 4.01]**

Heterogeneity: Tau² = 0.00; Chi² = 1.91, df = 3 (P = 0.59); I² = 0%
Test for overall effect: Z = 1.54 (P = 0.12)

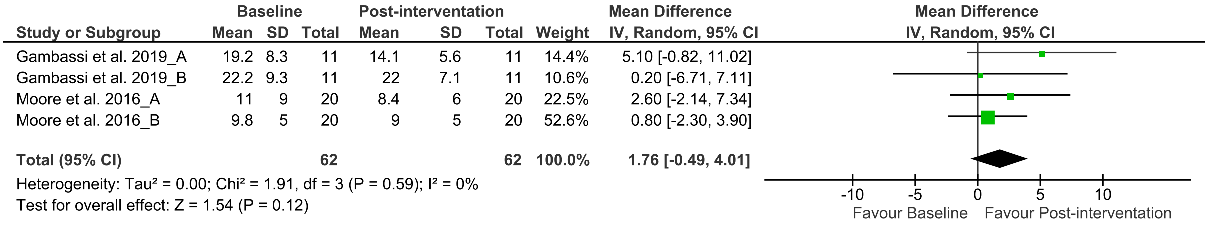

B

	Control			Intervention				Mean Difference
Study or Subgroup	Mean	SD	Total	Mean	SD	Total	Weight	IV, Random, 95% CI
Gambassi et al. 2019	-0.2	8.4	11	-5.1	7.3	11	28.0%	4.90 [-1.68, 11.48]
Moore et al. 2016	-0.8	5	20	-2.6	7.9	20	72.0%	1.80 [-2.30, 5.90]
**Total (95% CI)**			**31**			**31**	**100.0%**	**2.67 [-0.81, 6.14]**

Heterogeneity: Tau² = 0.00; Chi² = 0.61, df = 1 (P = 0.43); I² = 0%
Test for overall effect: Z = 1.50 (P = 0.13)

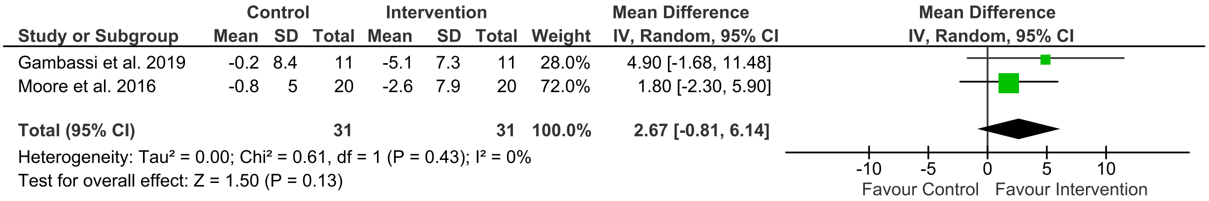

Supplementary file 12: Fasting blood glucose level changes (A) baseline to post-intervention, (B) difference in pre-and post-intervention at control and intervention groups after rehabilitation, (C) Funnel Plot. (SD: Standard deviation; IV: Inverse-variance, CI: Confidence interval; df: degree of freedom)

A

Study or Subgroup	Baseline Mean	Baseline SD	Baseline Total	Post-interventation Mean	Post-interventation SD	Post-interventation Total	Weight	Mean Difference IV, Random, 95% CI
Faulkner et al. 2013_A	5.91	1.48	30	5.69	1.41	30	2.2%	0.22 [-0.51, 0.95]
Faulkner et al. 2013_B	5.88	1.02	30	5.95	1.2	30	3.6%	-0.07 [-0.63, 0.49]
Fulkner et al. 2016_A	5.58	1.09	25	5.58	1.48	25	2.2%	0.00 [-0.72, 0.72]
Fulkner et al. 2016_B	6.45	1.77	22	6.57	1.61	22	1.2%	-0.12 [-1.12, 0.88]
Kirk et al. 2013_A	5.33	0.4	12	5.2	0.4	12	11.3%	0.13 [-0.19, 0.45]
Kirk et al. 2013_B	5.49	0.6	12	5.38	0.4	12	7.0%	0.11 [-0.30, 0.52]
MacKay-Lyons et al. 2022A	6.2	1.8	94	5.9	1.1	94	6.4%	0.30 [-0.13, 0.73]
MacKay-Lyons et al. 2022B	6	1.7	90	6	2.1	90	3.7%	0.00 [-0.56, 0.56]
Sakakibara et al. 2022_A	6	2	64	5.9	1.6	64	2.9%	0.10 [-0.53, 0.73]
Sakakibara et al. 2022_B	6	1.6	62	5.9	1.3	62	4.4%	0.10 [-0.41, 0.61]
Tang et al. 2013_A	5.3	1.2	22	5.1	1	22	2.7%	0.20 [-0.45, 0.85]
Tang et al. 2013_B	5.4	1.4	25	5	0.9	25	2.7%	0.40 [-0.25, 1.05]
Zou et al. 2015_A	5.11	0.51	28	4.77	0.33	28	22.9%	0.34 [0.12, 0.56]
Zou et al. 2015_B	5.03	0.44	28	5.01	0.35	28	26.7%	0.02 [-0.19, 0.23]
**Total (95% CI)**			**544**			**544**	**100.0%**	**0.15 [0.04, 0.26]**

Heterogeneity: Tau² = 0.00; Chi² = 6.76, df = 13 (P = 0.91); I² = 0%
Test for overall effect: Z = 2.72 (P = 0.007)

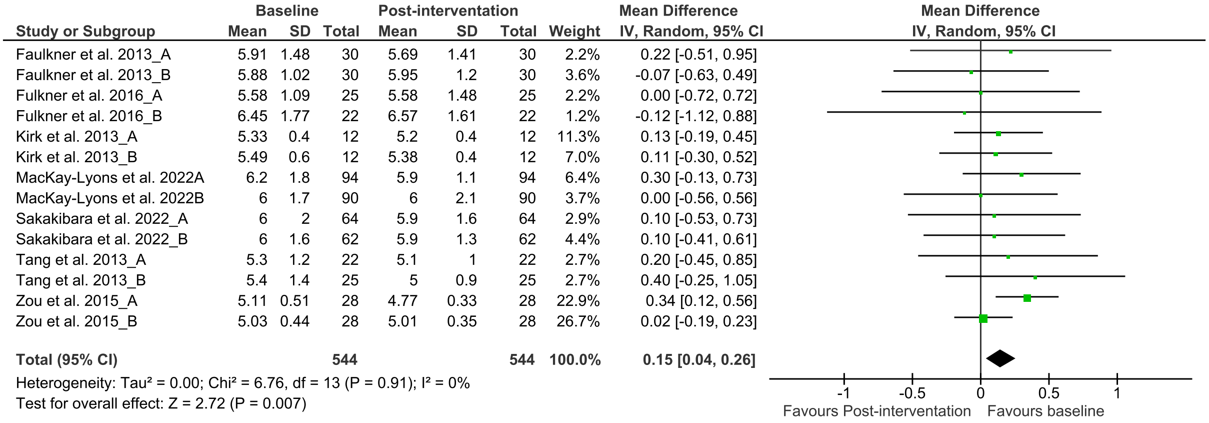

B

Study or Subgroup	Control Mean	Control SD	Control Total	Intervention Mean	Intervention SD	Intervention Total	Weight	Mean Difference IV, Random, 95% CI
Faulkner et al. 2013	-0.11	0.52	30	-0.13	0.4	30	34.0%	0.02 [-0.21, 0.25]
Fulkner et al. 2016	0.12	1.7	22	0	1.3	25	2.5%	0.12 [-0.75, 0.99]
Kirk et al. 2013	0	1.93	12	-0.3	1.57	12	0.9%	0.30 [-1.11, 1.71]
MacKay-Lyons et al. 2022	0.07	1.12	90	-0.22	1.44	94	13.6%	0.29 [-0.08, 0.66]
Sakakibara et al. 2022	-0.1	1.5	62	-0.1	1.8	64	5.6%	0.00 [-0.58, 0.58]
Tang et al. 2013	-0.4	1.1	25	-0.2	1.1	22	4.7%	-0.20 [-0.83, 0.43]
Zou et al. 2015	-0.02	0.4	28	-0.34	0.44	28	38.7%	0.32 [0.10, 0.54]
**Total (95% CI)**			**269**			**275**	**100.0%**	**0.17 [0.03, 0.30]**

Heterogeneity: Tau² = 0.00; Chi² = 5.45, df = 6 (P = 0.49); I² = 0%
Test for overall effect: Z = 2.38 (P = 0.02)

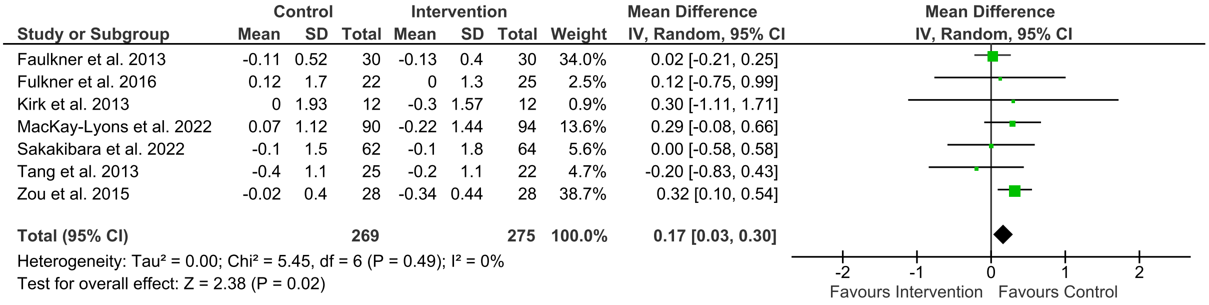

C

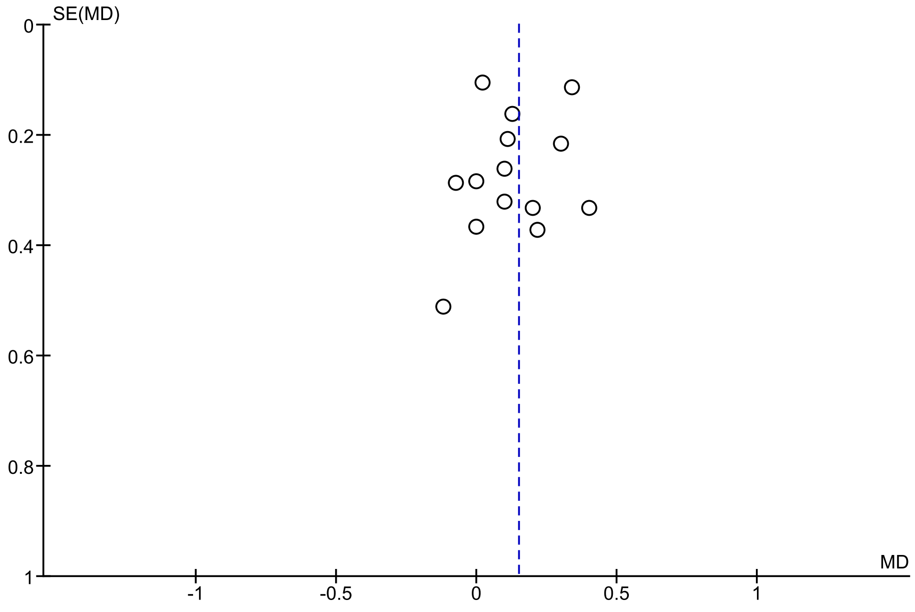

Supplementary file 13: Homocysteine level changes (A) baseline to post-intervention, and (B) difference in pre-and post-intervention at control and intervention groups after poststroke rehabilitation programs. (SD: Standard deviation; IV: Inverse-variance, CI: Confidence interval; df: degree of freedom)

A

	Control			Intervention				Mean Difference
Study or Subgroup	Mean	SD	Total	Mean	SD	Total	Weight	IV, Fixed, 95% CI
Sakakibara et al. 2022	-1.2	6.8	62	0.7	4.2	62	65.1%	-1.90 [-3.89, 0.09]
Tang et al. 2013	-2.7	5.1	25	-2.6	4.4	22	34.9%	-0.10 [-2.82, 2.62]
**Total (95% CI)**			**87**			**84**	**100.0%**	**-1.27 [-2.88, 0.33]**

Heterogeneity: Chi² = 1.10, df = 1 (P = 0.29); I² = 9%
Test for overall effect: Z = 1.55 (P = 0.12)

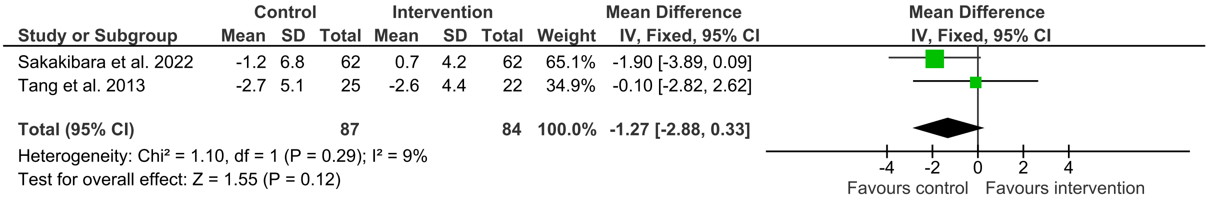

B

	Baseline			Post-intervention				Std. Mean Difference
Study or Subgroup	Mean	SD	Total	Mean	SD	Total	Weight	IV, Fixed, 95% CI
Sakakibara et al. 2022_A	12.5	3.7	64	13.2	4.6	62	37.0%	-0.17 [-0.52, 0.18]
Sakakibara et al. 2022_B	14.4	7.8	62	13.2	4.6	62	36.4%	0.19 [-0.17, 0.54]
Tang et al. 2013_A	13.4	4.9	22	10.8	3.7	22	12.4%	0.59 [-0.02, 1.19]
Tang et al. 2013_B	13.9	5.4	25	11.2	4.7	25	14.2%	0.52 [-0.04, 1.09]
**Total (95% CI)**			**173**			**171**	**100.0%**	**0.15 [-0.06, 0.37]**

Heterogeneity: Chi² = 6.90, df = 3 (P = 0.08); I² = 57%
Test for overall effect: Z = 1.41 (P = 0.16)

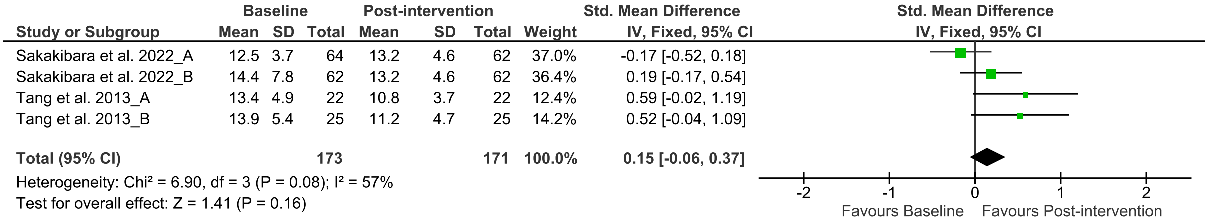

Supplement: Supplementary file 1 [file Datasheet1.pdf]
